# Identification of candidate *MLO* powdery mildew susceptibility genes in cultivated Solanaceae and functional characterization of tobacco *NtMLO1*

**DOI:** 10.1007/s11248-015-9878-4

**Published:** 2015-05-07

**Authors:** Michela Appiano, Stefano Pavan, Domenico Catalano, Zheng Zheng, Valentina Bracuto, Concetta Lotti, Richard G. F. Visser, Luigi Ricciardi, Yuling Bai

**Affiliations:** Laboratory of Plant Breeding, Wageningen University, Droevendaalsesteeg 1, 6708 PB Wageningen, The Netherlands; Department of Plant, Soil and Food Science, Section of Genetics and Plant Breeding, University of Bari Aldo Moro, Via Amendola 165/A, 70126 Bari, Italy; Institute of Biosciences and Bioresources, Italian National Research Council, Via Amendola 165/A, 70126 Bari, Italy; Department of Agro-Environmental Science, Chemistry and Crop Protection, University of Foggia, Via Napoli 25, 71100 Foggia, Italy; Institute of Vegetables and Flowers, Chinese Academy of Agricultural Sciences, No. 12 Zhongguan Cun Nan Da Jie, Beijing, 100081 China

**Keywords:** MLO, Solanaceae, Powdery mildew, Resistance, Plant breeding

## Abstract

Specific homologs of the plant *Mildew Locus O* (*MLO*) gene family act as susceptibility factors towards the powdery mildew (PM) fungal disease, causing significant economic losses in agricultural settings. Thus, in order to obtain PM resistant phenotypes, a general breeding strategy has been proposed, based on the selective inactivation of *MLO* susceptibility genes across cultivated species. In this study, PCR-based methodologies were used in order to isolate *MLO* genes from cultivated solanaceous crops that are hosts for PM fungi, namely eggplant, potato and tobacco, which were named *SmMLO1*, *StMLO1* and *NtMLO1*, respectively. Based on phylogenetic analysis and sequence alignment, these genes were predicted to be orthologs of tomato *SlMLO1* and pepper *CaMLO2*, previously shown to be required for PM pathogenesis. Full-length sequence of the tobacco homolog *NtMLO1* was used for a heterologous transgenic complementation assay, resulting in its characterization as a PM susceptibility gene. The same assay showed that a single nucleotide change in a mutated *NtMLO1* allele leads to complete gene loss-of-function. Results here presented, also including a complete overview of the tobacco and potato *MLO* gene families, are valuable to study *MLO* gene evolution in Solanaceae and for molecular breeding approaches aimed at introducing PM resistance using strategies of reverse genetics.

**Electronic supplementary material:**

The online version of this article (doi:10.1007/s11248-015-9878-4) contains supplementary material, which is available to authorized users.

## Introduction

Powdery mildew (PM) is a major fungal disease affecting thousands of plant species, caused by ascomycete fungi belonging to the order of Erysiphales (Glawe 2008). Chemical control of PM accounts for a large proportion of fungicides used in agricultural settings (Hewitt 1998). Therefore, the use of cultivars harbouring genetic sources of PM resistance is generally envisaged as a valuable strategy to reduce farming costs and to cope with public concerns related to environmental pollution and human health.

The *Mildew Locus O* (*MLO*) gene family encodes for plant-specific proteins harbouring several transmembrane domains, topologically reminiscent of metazoan G-protein coupled receptors (Devoto et al. 2003). Specific homologs of the *MLO* family act as susceptibility genes towards PM fungi. Indeed, their inactivation, through loss-of-function mutations or silencing, has been associated with a peculiar form of PM resistance, referred to as *mlo* resistance (Pavan et al. 2010). This is associated with the enhancement of exocytosis defence pathways at plant–pathogen interaction sites, which are thought to contribute to the prevention of fungal penetration into host cells (Assaad et al. 2004). Initially discovered in barley, *mlo* resistance has been later shown to occur in other plant species as well, specifically *Arabidopsis*, tomato, pea, pepper and bread wheat (Bai et al. 2008; Büschges et al. 1997; Consonni et al. 2006; Humphry et al. 2011; Pavan et al. 2011; Wang et al. 2014; Zheng et al. 2013). This eventually led to the formalization of a breeding approach based on the systematic inactivation of *MLO* susceptibility genes across cultivated species affected by the PM disease (Dangl et al. 2013; Pavan et al. 2010, 2011). Proof of concept for this strategy has been recently provided by the work of Wang et al. (2014), reporting the introduction of PM resistance in bread wheat following targeted mutagenesis of three *MLO* homoeoalleles. In contrast with most genetic sources of PM resistance, experimental data clearly indicate that *mlo* immunity is not specific towards particular fungal isolates and is extremely durable. For example, loss-of-function mutations of barley *HvMLO* confer resistance to all known isolates of the PM fungus *Blumeria graminis* f. sp. *hordei*, and is successfully employed in barley breeding since 1979 (Lyngkjaer et al. 2000). Similarly, pea *er1* PM resistance, originating from the loss of function of *PsMLO1*, was first reported more than 60 years ago and is the only resistance source worldwide used for breeding purposes (Harland 1948; Humphry et al. 2011; Pavan et al. 2013).

Following the completion of the respective genome sequencing projects, a number of *MLO* homologs variable between 12 and 19 has been identified in the diploid species Arabidopsis, rice, grapevine, peach, woodland strawberry and cucumber (Devoto et al. 2003; Feechan et al. 2008; Liu and Zhu 2008; Pessina et al. 2014; Schouten et al. 2014). Remarkably, when placed in MLO protein family phylogenetic trees, all dicot MLO isoforms experimentally shown to be required for PM susceptibility group in the same clade, referred to as clade V in scientific literature (e.g. Feechan et al. 2008; Pavan et al. 2011; Acevedo-Garcia et al. 2014). This shows that evolutionary studies on MLO proteins may predict candidates for being PM susceptibility factors.

Concerning solanaceous crops, we have functionally characterized the two *MLO* orthologs *SlMLO1* in tomato and *CaMLO2* in pepper, whose inactivation is causally associated with PM resistance (Bai et al. 2008; Zheng et al. 2013). In this work, we report the isolation, through a PCR-based approach, of three *MLO* genes from other cultivated Solanaceae, namely eggplant, potato and tobacco, which are likely to share a relation of orthology with *SlMLO1* and *CaMLO2*. The tobacco *MLO* homolog *NtMLO1* was chosen for a transgenic complementation assay, resulting in its functional characterization and identification of a loss-of-function mutant allele. Finally, newly available tobacco and potato genome sequences (Sierro et al. 2014; The Potato Genome Sequencing Consortium 2011) were exploited to provide a comprehensive overview of the *MLO* gene families in these species.

## Materials and methods

### PCR-based isolation and phylogenetic characterization of *MLO* putative orthologs

Young leaves of eggplant (*Solanum melongena* cv. Half Lange Violette), potato (*Solanum tuberosum* cv. Desiree) and tobacco (*Nicotiana tabacum* cv. Petit Havana SR1) were collected for RNA extraction, which was performed using the Trizol reagent (Invitrogen). After RNA purification with the NucleoSpin RNA II kit (Macherey–Nagel), cDNA was synthesized using the SuperScript III RT first-strand cDNA synthesis kit (Invitrogen) with oligo(dT) primers.

Aiming to identify sequences of *SlMLO1* putative orthologs, the primer pairs Sol-F1 (5′-CATTTGACATTTCCCCTTCTTC-3′)/Sol-R1 (5′-GCACCATGCATGAGTACCTCT-3′) and Sol-F2 (5′-TTGGCAGTTGCTCATGTATTG-3′)/Sol-R2 (5′-ATGGTGCCAGCTTCTAAGAG-3′) were designed on the untranslated and coding sequences of the *SlMLO1* gene (GeneBank accession number NM_001247885), respectively, (Primer3, Rozen and Skaletsky 2000) and used for PCR amplification of cDNAs. Amplicons obtained with the Sol-F2/Sol-R2 primer pair were purified using the NucleoSpin Extract II kit (Macherey–Nagel) and ligated (molar ratio 1:1) into the pGEM-T easy vector (Promega). Recombinant plasmids were cloned in *E. coli* DH10β chemically competent cells and recovered by using the Qiaprep spin miniprep kit (Qiagen). Sequencing reactions were performed using universal T7 and SP6 primers (Eurofins MWG Operon).

In order to obtain full-length coding sequences of potato and tobacco *MLO* genes, sequences overlapping with those of the amplicons above mentioned were retrieved by BLAST search, using the tomato *SlMLO1* coding sequence as query against expressed sequence tags (ESTs) and predicted coding sequence repositories, both available at the Sol Genomic Network (SGN) database (http://solgenomics.net), and then used for local alignment. The expression and sequence of candidate genes was verified by PCR amplification of cDNAs, using the primer pairs StMLO1-F (5′-ATGGCTAAAGAACGGTCG-3′)/StMLO1-R (5′-TTATTTGTTTCCAAAAGT-3′) and NtMLO1-F (5′-ATGGAGGCAACTCCGACTTG-3′)/NtMLO1-R (5′-TCAACTCATTTTGTTGCCAAATG-3′), cloning and sequencing, which were performed as above described.

In order to amplify a full-length *MLO* sequence in eggplant, the following primer pair was used: SmMLO1-F2 (5′-ATGGCTAAAGAACGGTCG-3′)/SmMLO1-R1 (5′-TTATTTGTTTCCAAAAGTAAAATCTGA-3′). The corresponding PCR product was cloned and sequenced as indicated above.

Full-length eggplant, potato and tobacco *MLO* genes (named *SmMLO1*, *StMLO1* and *NtMLO1*, respectively) were translated in silico. Corresponding protein sequences were used, together with those of dicot MLO proteins experimentally associated with PM susceptibility [*Arabidopsis thaliana* AtMLO2 (GenBank accession code NP172598), AtMLO6 (NP176350) and AtMLO12 (NP565902), *Solanum lycopersicum* SlMLO1 (NP001234814), *Capsicum annuum* CaMLO2 (AFH68055), *Pisum sativum* PsMLO1 (ACO07297), *Lotus japonicus* LjMLO1 (AAX77015) and *Medicago truncatula* MtMLO1 (ADV40949)] and those of the remaining twelve homologs of the *Arabidopsis thaliana* AtMLO protein family, for ClustalW alignment and the construction of a Unweighted Pair Group Method with Arithmetic Mean (UPGMA) phylogenetic tree. Bootstrap values were calculated from 100 replicates. All of these bioinformatic analyses were performed using the CLC sequence viewer software (http://www.clcbio.com/).

### Generation of transgenic plants overexpressing *NtMLO1*

Two different *NtMLO1* PCR products, differing for a single nucleotide polymorphism, were inserted into the Gateway-compatible vector pENTR D-TOPO (Invitrogen) and cloned in *E. coli* competent cells. Presence of the inserts was assessed by colony PCR, restriction enzyme digestion and sequencing using the universal M13 primer pair. Inserts were then transferred by LR recombination into the binary plasmid vector pK7WG2, harboring the 35S Cauliflower Mosaic Virus (CaMV) promoter for constitutive expression and the marker gene *nptII* for kanamycin resistance selection. Plasmids were inserted into *E. coli* competent cells and positive colonies were again screened by colony PCR and sequencing, as above. Recombinant vectors were finally extracted and transferred to the AGL1-*vir*G strain of *A. tumefaciens* by electroporation. A selected PM resistant tomato line, named Slmlo1, described by Bai et al. (2008) and carrying a loss-of-function deletion in the *SlMLO1* coding sequence, was used for transformation. This was performed according to the method described by McCormick et al. (1986). Briefly, seeds were surface-sterilized and sown on half-strength Murashige and Skoog (MS) agar supplemented with sucrose (10 g/l). Cotyledons were excised from 10-day-old seedlings, cut in two parts and submerged in an *A. tumefaciens* suspension with an OD_600_ value of about 0.125. Infected cotyledonary explants were placed abaxially on the GCF10 medium (4.3 g/l MS basal salt mixture, 8 g/l agar, 30 g/l sucrose, 108.73 mg/l Nitsch vitamins, 1.5 mg/l zeatin riboside, 0.2 mg/l indole-3-acetic acid, pH 5.8) supplemented with 1 ml/l acetosyringone at 25 °C for 48 h. Then, they were transferred to the GCF10 medium to which 100 mg/ml timentin and 50 mg/ml kanamycin were added and sub-cultured onto fresh medium every 3 weeks until shoot buds were observed. These were excised from the callus and transferred to the GCF11 medium (4.3 g/l MS basal salt mixture, 8 g/l agar, 30 g/l sucrose, 108.73 mg/l Nitsch vitamins, 1.9 mg/l zeatin riboside, pH 5.8) with 100 mg/ml timentin and 50 mg/ml kanamycin. After meristem development, the explants were transferred to the root-inducing medium MS30B5 (4.3 g/l MS basal salt mixture, 8 g/l agar, 30 g/l sucrose, 112 mg/l vitamin B5, 50 mg/ml kanamycin, pH 5.8). Once roots were developed, plantlets were finally located on woolen rock and grown in a greenhouse compartment.

For each of the two transformations with a different *NtMLO1* gene sequence, 20 T_1_ plants and two T_2_ families (each composed by fifteen individuals derived from self-pollination of individual T_1_ plants) were assayed for the presence of the construct, using the primer pair ntpIIF (5′-TCGGCTATGACTGGGCACAAC-3′)/ntpIIR (5′-AAGAAGGCGATAGAAGGCGA-3′), designed on the *ntpII* gene sequence, and the primer pair 35S-F (5′-GCTCCTACAAATGCCATCA-3′)/35S-R (5′-GATAGTGGGATTGTGCGTCA-3′), designed on the 35S promoter sequence. Expression of the transgene was assessed by qPCR using the primer pair NtMLO1_qFw (5′-GTGGAAATAAGTCCAGCATTATG-3′)/NtMLO1_qRev (5′-CACCCAAAGGTACGAGTACAATC-3′).

### Disease tests and *Oidium neolycopersici* quantification on transgenic plants

Three cuttings per T_1_ individuals and plants of the T_2_ families mentioned above were challenged with an isolate of the tomato PM fungus *Oidium neolycopersici* maintained at the Plant Breeding Department of the University of Wageningen, The Netherlands. The Slmlo1 mutant line and the susceptible cultivar Moneymaker (MM) were used as controls. Inoculation was performed as described by Pavan et al. (2008), by spraying plants with a suspension of conidiospores obtained from freshly sporulating leaves of heavily infected plants and adjusted to a final concentration of 4 × 10^4^ spores/ml. Inoculated plants were grown in a greenhouse compartment at 20 ± 2 °C with 70 ± 15 % relative humidity and day-length of 16 h. Disease evaluation was carried out 15 days after inoculation, based on a visual scoring as described by Bai et al. (2008) and/or analytically, by the relative quantification of the ratio between fungal and plant gDNAs. The latter was performed by the qPCR assay reported by Huibers et al. (2013). Specifically, plant and fungal genomic DNAs were extracted from *O. neolycopersici* infected tomato leaves (Qiagen DNeasy Plant Mini Kit) and used for amplification with the primer pairs On-F (5′-CGCCAAAGACCTAACCAAAA-3′)/On-R (5′-AGCCAAGAGATCCGTTGTTG-3′), designed on *O. neolycopersici* internal transcribed spacer (ITS) sequences (GenBank accession number EU047564), and Ef-F (5′-GGAACTTGAGAAGGAGCCTAAG-3′)/Ef-R (5′-CAACACCAACAGCAACAGTCT-3′), designed on the tomato *Elongation Factor 1α* (*Ef1α*) gene (Løvdal and Lillo 2009). Relative quantification was performed by the 2^–ΔΔCt^ method (Livak and Schmittgen 2001; Pfaffl 2001).

### In silico characterization of the tobacco and potato *MLO* gene families

In order to retrieve tobacco and potato MLO homologs, nucleotide sequences of *NtMLO1* and *StMLO1* and corresponding translated sequences were used as query for BLAST (BLASTn and tBLASTn) search against the Sol Genomics Network (SGN) and the Potato Genomics Resource (Spud DB) databases, using default parameters.

The number of transmembrane domains was predicted using the online software TMHMM (http://www.cbs.dtu.dk/services/TMHMM/). The putative number of introns was obtained using the online service FGENESH of Softberry (http://www.softberry.com/). Chromosomal localization and gene position of potato *MLO* genes were inferred by the annotations of the Potato Genome Consortium. Finally, the MEME (http://meme.nbcr.net/) (Bailey et al. 2009) package was used to predict functional motifs in the NtMLO and StMLO protein families. Predicted tobacco NtMLO and potato StMLO proteins were used to integrate the phylogenetic tree described in the previous section, according to the same methodologies above mentioned.

## Results

### Identification of *MLO* gene sequences from cultivated Solanaceae

Two primer pairs, one designed on the untranslated sequence and the other on the coding sequence of tomato *SlMLO1*, were used to amplify homologous sequences from eggplant, potato and tobacco cDNAs. PCRs performed with the Sol-F1/R1 primer pair failed, thus suggesting the occurrence of polymorphic sequences in untranslated regions. In contrast, PCR performed with the Sol-F2/R2 primer pair, designed within the *SlMLO1* coding sequence, resulted in single amplification products of 876 bp. Full-length sequences of a 1560 bp tobacco gene, named *NtMLO1,* and a 1557 bp potato gene, named *StMLO1*, were obtained by assembling partial gene sequences of PCR products with overlapping sequences retrieved by the interrogation of the SGN database. Amplification and sequencing of *StMLO1* and *NtMLO1* from potato and tobacco cDNAs provided evidence for their actual expression in leaves and validated their sequences. These were deposited in the GenBank database with the accession codes KM244715 (*StMLO1*) and KM244716 (*NtMLO1*).

In order to clone an eggplant *MLO* gene putatively involved in PM susceptibility, several primers were designed, based on the identification of conserved regions from the alignment of *SlMLO1*, *StMLO1* and *NtMLO1*. These primers were then tested on eggplant cDNA. The SmMLO1-F2/SmMLO1-R1 primer pair produced a single PCR amplification product. The corresponding sequence of 1572 bp was named *SmMLO1* and deposited in the GenBank database with the accession code KM244717.

### Bioinformatic analyses support the identification of solanaceous MLO functional orthologs required for PM susceptibility

**Fig. 1:**
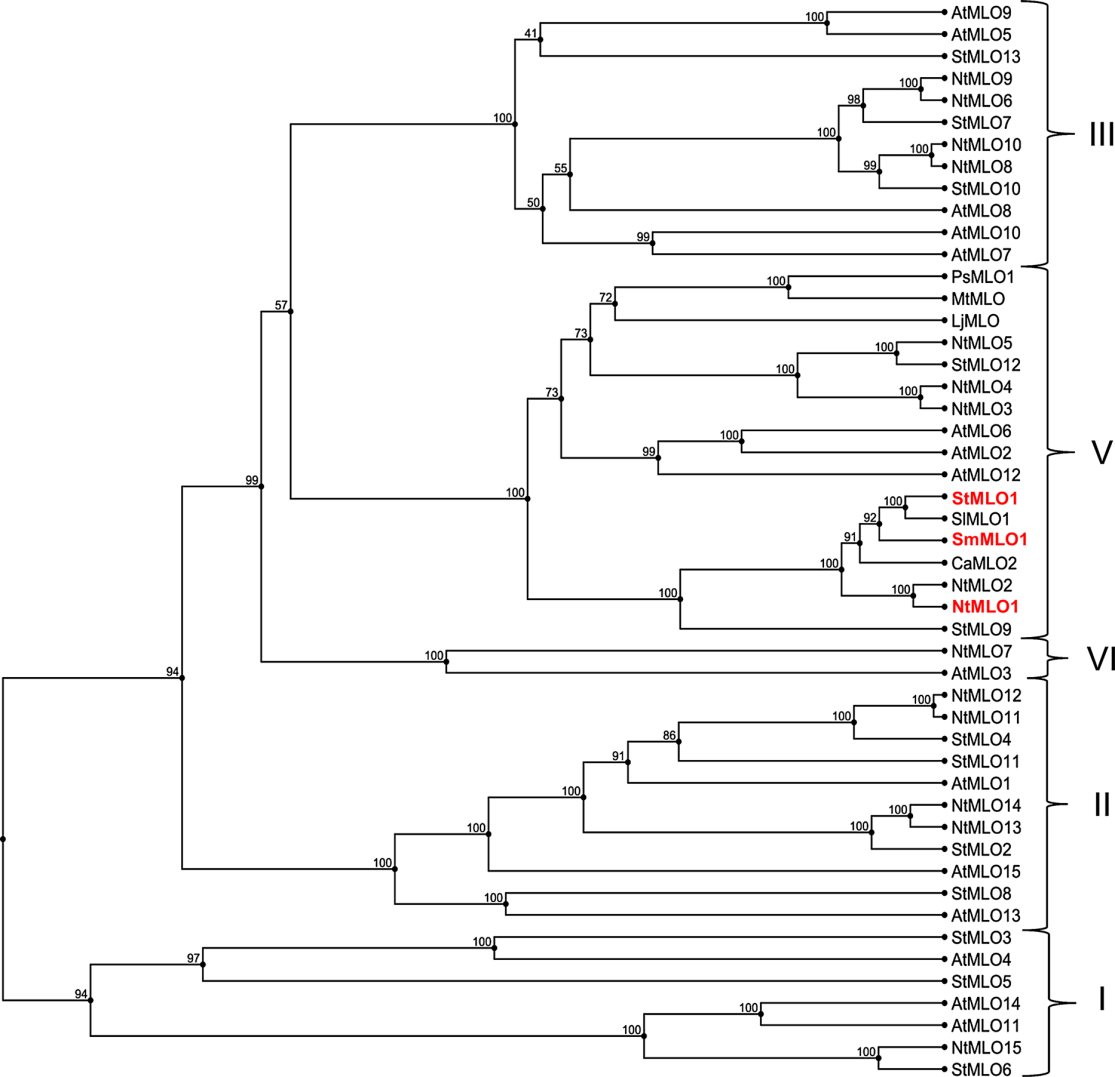
UPGMA-based tree of full-length MLO proteins. The dataset includes the tobacco NtMLO, potato StMLO and Arabidopsis AtMLO protein families, tomato SlMLO1, pepper CaMLO2, eggplant SmMLO1, pea PsMLO1, lotus LjMLO1 and barrel clover MtMLO1. Phylogenetic clades are designated with *Roman numbers* based on the position of AtMLO homologs, according to the nomenclature indicated by Feechan et al. (2008). Homologs identified by means of a PCR-based approach in this study (SmMLO1, StMLO1 and NtMLO1) are indicated in bold red. Numbers at each node represent bootstrap support values (out of 100 replicates)

StMLO1, NtMLO1 and SmMLO1 protein sequences were used to perform a phylogenetic analysis. With strong bootstrap support, they were found to group in the phylogenetic clade V, containing all the dicot MLO homologs so far experimentally shown to be required for PM susceptibility (AtMLO2, AtMLO6, AtMLO12, SlMLO1, CaMLO2, PsMLO1, LjMLO1 and MtMLO1) (Fig. 1), thus indicating they could possibly be functionally related.

Previous studies highlighted the presence of amino acid residues highly conserved either in the whole MLO protein family or in MLO orthologs involved in the interaction with PM fungi, which are predicted to play a key functional role (Elliott et al. 2005; Panstruga 2005). All of these residues were found to be present in the StMLO1, NtMLO1 and SmMLO1 protein sequences (Supplementary Fig. 1), providing further evidence for the identification of *MLO* genes required for PM susceptibility.

Finally, another strong bioinformatic indication for the identification of solanaceous *MLO* susceptibility genes was provided by aligning the coding sequences of *StMLO1*, *NtMLO1* and *SmMLO1* with those of the PM susceptibility genes *SlMLO1* and *CaMLO2*, functionally characterized in tomato and pepper, respectively (Bai et al. 2008; Zheng et al. 2013) (Supplementary Fig. 2). Indeed, this revealed a very high percentage of nucleotide identity (81.4 % between tomato and tobacco, 87.5 % between tomato and eggplant and 94.8 % between tomato and potato), suggesting that all of these solanaceous *MLO* genes are orthologs.

### Tobacco *NtMLO1* complements tomato *SlMLO1* in a functional complementation assay

In order to characterize *NtMLO1* at the functional level, we set up an assay based on its transgenic overexpression in the previously described tomato line Slmlo1, which carries a loss-of-function mutation in the tomato *SlMlo1* homolog and is thus resistant to the PM fungus *O. neolycopersici* (Bai et al. 2008). We hypothesised that overexpression of *NtMLO1* would have restored PM susceptibility in the tomato Slmlo1 mutant line, thereby demonstrating functional conservation between *NtMLO1* and *SlMLO1*.

**Fig. 2:**
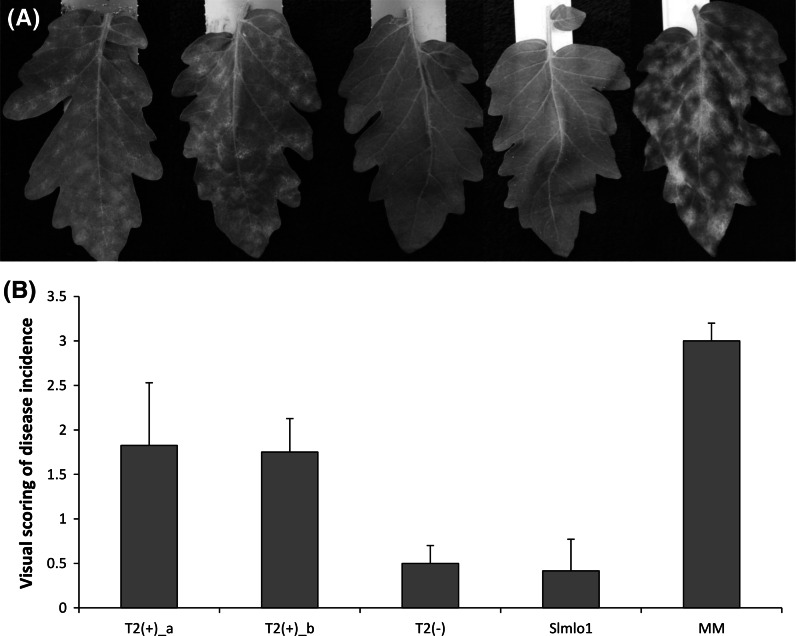
Effects of the transgenic expression of *NtMLO1* in a tomato *mlo* loss-of-function genetic background. **a** From left to right as follows: one individual of a T_2_ family positive for the presence the *NtMLO1* overexpression construct [T_2__a(+)]; one individual of another independent T_2_ family positive for the presence of the *NtMLO1* overexpression construct [T_2__b(+)]; one T_2_ individual negative for the presence of the overexpression construct [T_2_(−)]; one individual of the tomato Slmlo1 mutant line, carrying a loss of function deletion in the *SlMLO1* gene; one individual of the susceptible cultivar Moneymaker (MM). **b** Reports the average visual scoring of disease incidence observed on the individuals of the same two T_2_ families [T_2__a(+) and T_2__b(+)]; individuals of both T_2__a and T_2__b families negative for the presence of the 35S::*NtMLO1* construct [T_2_(−)]; individuals of the Slmlo1 mutant line; individuals of the cultivar MM. The scale from 0 (completely resistant) to 3 (fully susceptible) reported by Bai et al. (2008), was used for scoring. *Bars* and *standard errors* refer to 11 T_2_(+)_a plants, 10 T_2_(+)_b plants, 9 T_2_(−) plants, 10 Slmlo1 plants and 10 MM plants

After transformation, cuttings of 20 T_1_ transgenic individuals were challenged with *O. neolycopersici*. Fifteen of the tested T_1_ individuals showed restoration of PM symptoms (data not shown). In order to confirm this result, two T_2_ families of the fifteen individuals (T_2__a and T_2__b) derived from self-pollination of two different T_1_ plants were also inoculated, together with MM (the susceptible control) and the Slmlo1 mutant line (the resistant control). The presence of the overexpression construct in segregating T_2_ families was assessed by PCR amplification with primer pairs designed on the *nptII* gene and the 35S promoter (Supplementary Fig. 3). T_2_ individuals not carrying the overexpression construct [T_2_(−)], as well as individuals of the Slmlo1 mutant line, showed no *NtMLO1* expression and an average of disease score of about 0.5. In contrast, T_2_ individuals of the two families positive for the presence of the construct [T_2_(+)_a and T_2_(+)_b] showed *NtMLO1* expression and an average disease score of 1.8 and 1.7, respectively (Fig. 2 and Supplementary Fig. 4).

### A *NtMLO1* point mutation causing the substitution of a conserved glutamine residue results in gene loss of function

**Fig. 3:**
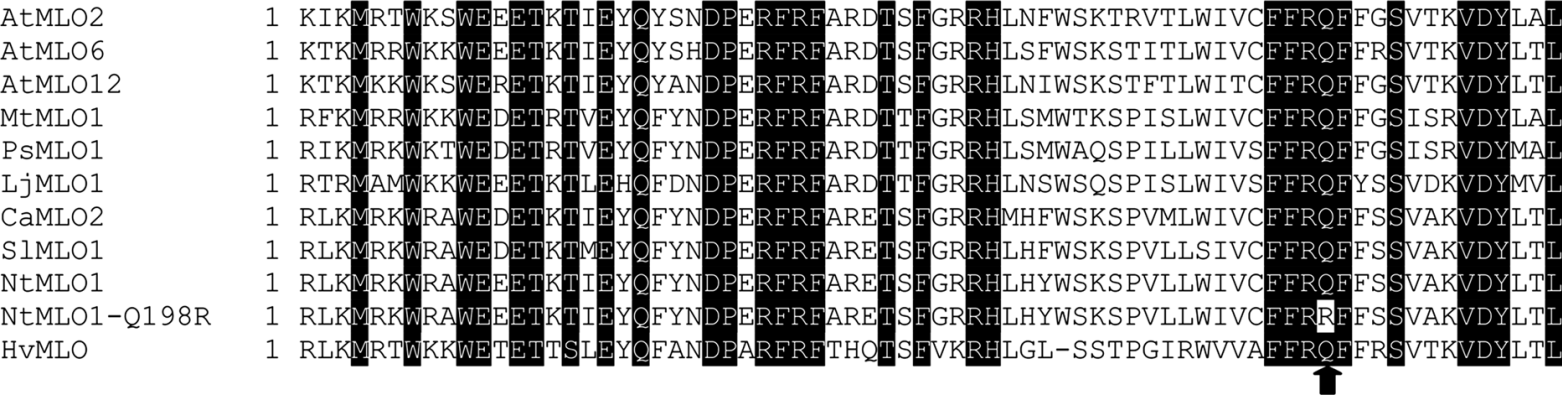
Alignment of part of the second MLO intracellular loop from several MLO proteins experimentally shown to be required for powdery mildew susceptibility (*Arabidopsis* AtMLO2, AtMLO6 and AtMLO12, tomato SlMLO1, pepper CaMLO2, pea PsMLO1, lotus LjMLO1, barrel clover MtMLO1 and barley HvMLO), and NtMLO1 proteins derived from the conceptual translation of the two inserts obtained during the cloning procedure (NtMLO1 and NtMLO1-Q198R). The latter is characterized by the substitution of an invariable glutamine with arginine, whose position is indicated by an arrow

**Fig. 4:**
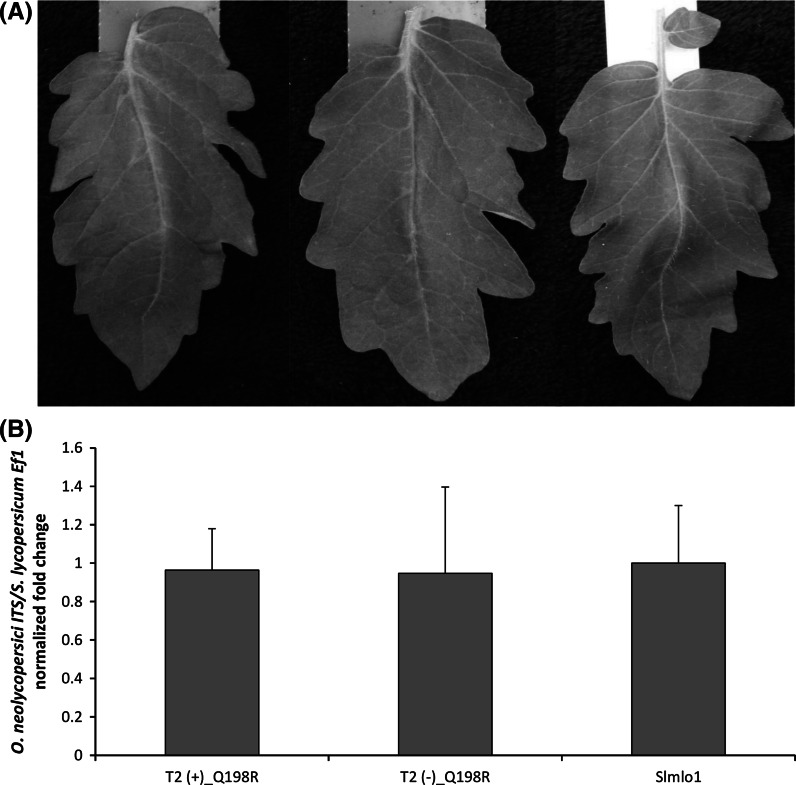
Effects of the transgenic expression of a *NtMLO1* mutant sequence, resulting in the substitution of a glutamine residue with arginine in the protein second intracellular loop (Q198R). **a** The phenotype of a plant of the tomato loss-of-function Slmlo1 line (*right*) and transgenic individuals from two different T_2_ families (*left* and* centre*) assessed for transgene overexpression. **b** The relative quantification of the ratio between *Oidium neolycopersici* and plant gDNAs in transgenic individuals of the same T_2_ families assessed for the presence or absence of the overexpression construct [T_2_(+)_Q198R and T_2_(−)_Q198R, respectively] and in the tomato Slmlo1 mutant line. *Bars* and *standard errors* refer to 11 and 7 transgenic individuals for NtMLO1_Q198R-a and b, respectively, and 10 Slmlo1 plants

During the preparation of the 35S::*NtMLO1* overexpression vector, we accidentally cloned another insert, carrying a single nucleotide polymorphism in the tobacco *NtMLO1* gene. This resulted in the substitution of a glutamine residue, located in the protein second intracellular loop and previously reported to be invariable throughout the whole MLO protein family, with arginine (Q198R, Fig. 3). We could not get the same arginine-coding insert by repeating the cloning procedure several times from tobacco cDNA, so we assumed that this resulted from a mutation due to an error by the Taq polymerase used for amplification. Nonetheless, in order to study the effect of this substitution on protein function, we developed transgenic lines carrying an overexpression construct for this insert. Following *O. neolycopersici* inoculation, none of 20 individual T_1_ plants developed disease symptoms. Individuals of two independent T_2_ families positive for the presence of the construct [T_2_(+)_Q198R-a and b] were found to express the transgene, as assessed by qPCR (Supplementary Fig. 4). Nevertheless, following *O. neolycopersici* challenge, no PM symptoms were visible on [T_2_(+)_Q198R] individuals, which were phenotypically undistinguishable from those of the Slmlo1 line (Fig. 4a). In order to test whether the mutated *NtMLO1* sequence maintained some residual functional activity, even so still resulting in a macroscopically resistant phenotype, we quantified, in transgenic individuals of the two T_2_ families, the relative fold-change of the ratio between *O. neolycopersici* and tomato gDNAs. Compared to the Slmlo1 line, no significant difference was found (Fig. 4b), indicating that the point nucleotide mutation causing the substitution of glutamine with arginine in the NtMLO1 protein sequence leads to complete gene loss of function.

### In silico characterization of tobacco and potato *MLO* families

Recently released sequences from potato (group *Phureja DM1*) and tobacco (cv. *Basma Xanthi*) prompted us to perform a genome-wide search aiming to characterize the *MLO* gene families in these species. This search revealed a total of 15 and 13 predicted tobacco *NtMLO* and potato *StMLO* loci, respectively, which were named according to the nomenclature specified in Supplementary Tables 1 and 2. A predicted tobacco coding sequence, referred to as mRNA_127718_cds in the Sol Genomics Database, was found to be identical to *NtMLO1*. No sequence fully matching with *StMLO1* could be identified by the interrogation of the Potato Genomics Resource database, but in its place a partial gene sequence showing 100 % of identity with the same gene.

For tobacco and potato MLO proteins, amino acid length and number of transmembrane domains were inferred (Supplementary Table 1 and Supplementary Table 2). In addition, information on chromosomal localization and intron number was available for predicted *StMLO* genes (Supplementary Table 2).

The tobacco NtMLO and potato StMLO protein families were used as input to search for conserved motifs, using an approach similar to the one previously reported by Deshmukh et al. (2014). We looked for motifs with length ranging from 40 to 70 residues and shared by at least three homologs. For each of the two families, seven motifs were identified. Of these, five were found to be at least partially matching with those identified in the soybean protein family (Deshmukh et al. (2014) (Supplementary Table 3).

A comparative analysis was carried out in order to establish phylogenetic relationships between the NtMLO and the StMLO protein families and MLO proteins from other dicot plant species. The analysis resulted in the distinction of five clades, designated with Roman numbers based on the position of Arabidopsis AtMLO homologs, according to the nomenclature indicated by Feechan et al. (2008) (Fig. 1). Besides NtMLO1 and StMLO1, additional NtMLO (NtMLO2, NtMLO3, NtMLO4 and NtMLO5) and StMLO (StMLO9 and StMLO12) homologs were found to group in clade V together with all dicot MLO proteins previously associated with PM susceptibility.

## Discussion

In previous studies, we functionally characterized tomato *SlMLO1* and pepper *CaMLO2* as two solanaceous *MLO* susceptibility genes, as their inactivation was causally associated with PM resistance (Bai et al. 2008; Zheng et al. 2013). Starting from this information, we followed here a combined approach based on database search and PCR amplification, which resulted in the isolation of three *MLO* genes from other widely distributed solanaceous species affected by the PM disease, namely eggplant (*SmMLO1*), potato (*StMLO1*) and tobacco (*NtMLO1*). PM disease represents one of the most important fungal diseases of tobacco and eggplant (Bubici and Cirulli 2008; Darvishzadeh et al. 2010) and in conducive environments may lead to important economic losses in potato cultivation (Glawe et al. 2004).

A chain of evidence, based on phylogenetic relatedness (Fig. 1) and sequence conservation with other known PM susceptibility genes and proteins (Supplementary Fig. 1 and Supplementary Fig. 2) was provided, suggesting the identification of solanaceous orthologs of *SlMLO1* and *CaMLO2*. Aiming at the functional characterization of *NtMLO1*, we set up an assay based on its heterologous overexpression in a tomato *mlo*-mutant genetic background, taking advantage from the availability of a tomato resistant line and routine protocols for tomato genetic transformation (Bai et al. 2008). Success of such an assay, as demonstrated by the restoration of symptoms in transgenic plants (Fig. 2), provides a final evidence for the role of *NtMLO1* as a PM susceptibility gene. Although it was not proven at the functional level, we speculate that both *StMLO1* and *SmMLO1* are involved in PM susceptibility in potato and eggplant, as they are, at the nucleotide level, even closer than *NtMLO1* to *SlMLO1* and *CaMLO2*.

While completing this work, newly released sequences of potato and tobacco became available. Thus, a genome-wide search was performed, which allowed to retrieve additional *MLO* homologs and, presumably, to characterize the complete tobacco and potato *MLO* gene families. Phylogenetic analysis using these sequences highlighted the presence of additional NtMLO and StMLO proteins in clade V, previously shown to group dicot MLO homologs acting as PM susceptibility factors (Fig. 1). Functional redundancy of MLO homologs belonging to this clade has been shown to occur in *Arabidopsis thaliana*, as the simultaneous inactivation of the three homolog genes *AtMLO2*, *AtMLO6* and *AtMLO12* is required to result in complete PM resistance. Thus, functional analyses, such as the transgenic complementation test above mentioned, might lead to the identification of additional solanaceous MLO homologs playing a role in the interaction with PM fungi.

Interestingly, due to a polymerase error during the cloning procedure, we also had the opportunity to verify the crucial role of a glutamine residue localized in the second intracellular MLO domain. This amino acid has been shown to be invariable throughout the whole MLO protein family and therefore predicted to be fundamental for the role of MLO proteins as PM susceptibility factors (Elliott et al. 2005). Indeed, its replacement with arginine in tobacco NtMLO1 (Fig. 3) resulted in complete failure of transgenic complementation, as inferred by visual scoring and relative quantification of fungal gDNA with respect to plant gDNA (Fig. 4). This result represents a complement to earlier investigations addressed to the functional characterization of MLO proteins (Reinstädler et al. 2010; Pavan et al. 2013).

A growing body of experimental evidence supports the view that *mlo*-based resistance can be conveniently pursued as a strategy to cope with the PM disease in practical breeding (Pavan et al. 2010). Therefore, we predict that results here provided might be of great interest for future activities aimed at the introduction of PM resistance in Solanaceae. Targeted identification of mutations of *MLO* susceptibility genes can be achieved through conventional approaches of TILLING (targeted induced local lesions in genomes) or RNA interference (McCallum et al. 2000; Matthew 2004). In addition, cutting-edge technologies of genome editing are also available to the breeder, based on zinc finger nucleases (ZFNs), clustered regularly interspaced short palindromic repeat (CRISPR) and transcription activator-like effector nucleases (TALEN) (Gaj and Gersbach 2013; Terns and Terns 2014). Noteworthy, a TALEN-based approach has been recently successfully applied to introduce PM resistance in bread wheat through simultaneous targeting of three homoelog *MLO* alleles, as mentioned in Wang et al. (2014).

## Electronic supplementary material

Supplementary material 1 (DOCX 1431 kb)
