# Supplementary material for: Identification of candidate MLO powdery mildew susceptibility genes in cultivated Solanaceae and functional characterization of tobacco NtMLO1
Source: Transgenic Res. 2015 May 7;24(5):847–58. doi: 10.1007/s11248-015-9878-4 (PMC4569668; doi:10.1007/s11248-015-9878-4)
Supplement: Supplementary file 1 — Supplementary material 1 (DOCX 1431 kb) [file 11248_2015_9878_MOESM1_ESM.docx]

**Electronic Supplementary Material**

**Transgenic Research**

**Identification of candidate *MLO* powdery mildew susceptibility genes in cultivated Solanaceae and functional characterization of tobacco *NtMLO1***

Michela Appiano^1,^**^†^**, Stefano Pavan^2,^**^†^**, Domenico Catalano^3^, Zheng Zheng^5^, Valentina Bracuto^2^, Concetta Lotti^4^, Richard G. F. Visser^1^, Luigi Ricciardi^2^, Yuling Bai^1,*^

^1^Laboratory of Plant Breeding, Wageningen University, Droevendaalsesteeg 1, 6708 PB Wageningen, The Netherlands

^2^Department of Plant, Soil and Food Science, Section of Genetics and Plant Breeding, University of Bari Aldo Moro, via Amendola 165/A, 70126 Bari, Italy

^3^Institute of Biosciences and Bioresources, Italian National Research Council, via Amendola 165/A, 70126 Bari, Italy

^4^Department of Agro-Environmental Science, Chemistry and Crop Protection, University of Foggia, via Napoli 25, 71100 Foggia, Italy

^5^Institute of Vegetables and Flowers, Chinese Academy of Agricultural Sciences, No. 12 Zhongguan Cun Nan Da Jie, 100081, Beijing, China

**^†^**these authors equally contributed to this work

^*^ corresponding author

e-mail: bai.yuling@wur.nl

tel: +31 317 482136

fax: +31 317 483457

**Supplementary Fig. 1** Protein multiple alignment of a dataset composed of eggplant SmMLO1, potato StMLO1, tobacco NtMLO1, the fifteen Arabidopsis AtMLO homologs and the susceptibility proteins SlMLO1 (tomato), CaMLO2 (pepper), PsMLO1 (pea), LjMLO1 (lotus) and MtMLO1 (barrel clover). Black shading shows amino acid residues reported to be conserved throughout the whole MLO protein family, whereas grey shading highlights residues shared by MLO proteins experimentally shown to be required for powdery mildew susceptibility

MtMLO1 1 ------------------------------MAEDKVYERTLEETPTWAVAVVCFVLLAIS
PsMLO1 1 ------------------------------MAEEGVKERTLEETPTWAVAVVCLVLLAVS
LjMLO1 1 --------------------------------MDKVAQKKLEETPTWAVAVVCFVMLAIS
AtMLO2 1 -------------------------------MADQVKERTLEETSTWAVAVVCFVLLFIS
AtMLO6 1 -------------------------------MADQVKEKTLEETSTWAVAVVCFVLLLIS
AtMLO12 1 ---------------------------------MAIKERSLEETPTWAVAVVCFVLLFIS
StMLO1 1 ----------------------------------MAKERSMEATPTWAIAVVCFILLAIS
SlMLO1 1 ----------------------------------------MEATPTWAIAVVCFILLAIS
SmMLO1 1 ----------------------------------MAKERSMEGTPTWAVAVVCFILLAIS
CaMLO2 1 ----------------------------------MAKERSMEATPTWAVAVVCFILLAIS
NtMLO1 1 ----------------------------------------MEATPTWAVAAVCFILLAIS
AtMLO7 1 --------MITRSRCRRSLLWFLVFHGGATATGAPSGGKELSQTPTWAVAVVCTFLILIS
AtMLO10 1 -----MATRCFWCWTTLLFCSQLLTGFARASSAGGAKEKGLSQTPTWAVALVCTFFILVS
AtMLO8 1 MGIIDGSLLRRLICLCLWCLLGGGVTVVTAEDEKKVVHKQLNQTPTWAVAAVCTFFIVVS
AtMLO5 1 ------------------------MAGGGGGSTSGEGPRELDQTPTWAVSTVCGVIILIS
AtMLO9 1 -------------------------MAGGGGGGGGEGPRQLDQTPTWAVSTVCGVIILIS
AtMLO3 1 ----------------------MTDKEESNHSSEVGAVRSLQETPTWALATVCFFFIAVS
AtMLO11 1 -----------------------MGEGEENGNEADSNERSLALSPTWSVAIVLTVFVVVS
AtMLO14 1 -----------------------------MREETEPSERTLGLTPTWSVATVLTIFVFVS
AtMLO4 1 ------------------------------MEHMMKEGRSLAETPTYSVASVVTVLVFVC
AtMLO1 1 -------------------------------MGHGGEGMSLEFTPTWVVAGVCTVIVAIS
AtMLO15 1 ---------------------------------MAGGGTTLEYTPTWVVALVCSVIVSIS
AtMLO13 1 --------------------------------MAEARSGSLEYTPTWVVAFICFIIVLLS

MtMLO1 31 IVIEHIIHAIGKWFKKKNKNALYEALEKVKGELMLMGFISLLLTVFQDYISKICISEKVG
PsMLO1 31 ILIEHIIHVIGKWLKKRNKNALYEALEKIKGELMLLGFISLLLTVFQDNISKICVSQKIG
LjMLO1 29 IIIEHGIEAIEKWLEKRHKKALHEAVEKIKGELMLMGFISFLLTVFKDPISNICISKQVA
AtMLO2 30 IVLEHSIHKIGTWFKKKHKQALFEALEKVKAELMLLGFISLLLTIGQTPISNICISQKVA
AtMLO6 30 IVIEKLIHKIGSWFKKKNKKALYEALEKVKAELMLMGFISLLLTIGQGYISNICIPKNIA
AtMLO12 28 IMIEYFLHFIGHWFKKKHKKALSEALEKVKAELMLLGFISLLLVVLQTPVSEICIPRNIA
StMLO1 27 IFIEQIIHHIGEWLLEKRKKPLYEALEKIKAELMLLGFLSLLLTVLQEPVSNLCVPKSIG
SlMLO1 21 IFIEQIIHHIGEWLLEKRKKSLYEALEKIKAELMLLGFLSLLLTVLQDPVSNLCVPKSVG
SmMLO1 27 IFIEQIIHHLGEWLLEKHKKPLHEALEKIKAELMLLGFISLLLTVVQDPVSNICVPKTVG
CaMLO2 27 IXIEQIMHHLGEWLLKKHKKPLYEALEKIKAELMLLGFISLLLTVIQDPVSNLCVPKSVG
NtMLO1 21 IFIEQIIHHLGEWLLKKHKKPLYEALEKIKAELMLLGFISLLLTVVQSPVSNLCVPKSVG
AtMLO7 53 HLLEKGLQRLANWLWKKHKNSLLEALEKIKAELMILGFISLLLTFGEPYILKICVPRKAA
AtMLO10 56 VLLEKALHRVATWLWEKHKNSLLEALEKIKAELMILGFISLLLTFGEQYILKICIPEKAA
AtMLO8 61 VLLEKLLHKVGKVLWDRHKTALLDALEKIKAELMVLGFISLLLTFGQTYILDICIPSHVA
AtMLO5 37 IVLELMIHKIGEVFTERRKKALYEALQKIKNELMVLGFISLLLTFGQNYIASLCVASRYG
AtMLO9 36 IILELIIHKVGEVFERKKKKALFEALEKIKNELMVLGFISLLLTFGQNYIASICVPSRYG
AtMLO3 39 ICLERLINLLSTRLKKNRKTSLLEAVEKLKSVLMVLGFMSLMLNVTEGEVSKICIPIKYA
AtMLO11 38 LIVERSIYRLSTWLRKTKRKPMFAALEKMKEELMLLGFISLLLTATSSTIANICVPSSFY
AtMLO14 32 LIVERSIHRLSNWLQKTKRKPLFAALEKMKEELMLLGFISLLLTATSSTIANICVSSSFH
AtMLO4 31 FLVERAIYRFGKWLKKTRRKALFTSLEKMKEELMLLGLISLLLSQSARWISEICVNSSLF
AtMLO1 30 LAVERLLHYFGTVLKKKKQKPLYEALQKVKEELMLLGFISLLLTVFQGLISKFCVKENVL
AtMLO15 28 FAVERLIHRAGKHFKNNDQKQLFGALQKIKEELMLVGFISLLLSVGQSKIAKICISKELS
AtMLO13 29 LLAERGLHHLGKCLKRRQQDALFEALQKLKEELMLLGFISLMLTVSQAAIRHICVPPALV

MtMLO1 91 STWHP-CSTPKTKTASNDENS--------ESENHDRKLLEYFDPNPRRILATKGYDQCADK
PsMLO1 91 STWHP-CSTSNTKAKAKSDESL------DYKTNNDRKLLEYFDPIPRRILATKGYDKCFDK
LjMLO1 89 STWHP-CHPEEKKKGPEG-----------------------------------YYDKCAKD
AtMLO2 90 STMHP-CSAAEEAKKYGKKDAGKKDDGDGDKPGRRLLLELAESYIHRRSLATKGYDKCAEK
AtMLO6 90 ASMHP-CSASEEARKYGKKDVPKEDE---EENLRRKLLQLVDSLIPRRSLATKGYDKCAEK
AtMLO12 88 ATWHP-CSNHQEIAKYGKDYID-------DGRKILEDFDSNDFYSPRRNLATKGYDKCAEK
StMLO1 87 YSWHP-CKPKADAQ------------------------------------SEYEVTSCDKK
SlMLO1 81 YSWHP-CMAKEDAK------------------------------------SEYD-DPCLPK
SmMLO1 87 YSWHP-CKAQEDDK------------------------------------PKYD-DPCLEK
CaMLO2 87 YSWHP-CKADEDVK------------------------------------SEYD-DPCLQK
NtMLO1 81 YSWHP-CKSDEAAK------------------------------------NKYD-DPCLPK
AtMLO7 113 LSMLP-CLSEDTVL------------FQKLAP--SSLSRHLL-------AAGDTSINC-KQ
AtMLO10 116 ASMLP-CPAPSTHD------------QDKT-------HRRRL-------AAATTSSRC-DE
AtMLO8 121 RTMLP-CPAPNLKK------------EDDDNG--ESHRRLLSFEHRFLSGGEASPTKCTKE
AtMLO5 97 HAMSF-CGPYDGPS------------GESKKP-------KTTEHLERRVLADAAPAQC-KK
AtMLO9 96 HAMSF-CGPYDGPS-----------EDDRKKLKKTDHAMRILYSVQRRSLADAPPVNC-KK
AtMLO3 99 NRMLP-CRKTIKSHNDVSEDDDDDDGDNH---------------------DNSFFHQCSSK
AtMLO11 98 NDRFLPCTRSEIQ---------EELESG-STVKRNLLTKSLFFNIFRRRLDVIKRTTC-SE
AtMLO14 92 NDRFVPCTPSEIN---------EELESTISTVKRTQLTRSLFLHTLRRRLSGIGEDTC-SE
AtMLO4 91 NSKFYICSEEDYG---------IHKKV------LLEHTSSTNQSSLPHHGIHEASHQC-GH
AtMLO1 90 MHMLP-CSLDSRREAGASEHKNVTAKEHFQTFLPIVGTTRR----LLAEHAAVQVGYCSEK
AtMLO15 88 EKFLP-CTKPAGAEKSLKDSSHFQ-----------FSFTGR----HLLAGDAPAGDYCSLK
AtMLO13 89 NNMFP-CKKPLEEHHAPKSSHSI--------------INNA----RHLLSTGESPDHCAAK

MtMLO1 143 G--KVALVSAYGIHELHIFIFVLAIFHILQCIITLALGRFKMRRWKKWEDETRTVEYQFYN-
PsMLO1 145 G--QVALVSAYGIHQLHIFIFVLALFHILQCIITLTLGRIKMRKWKTWEDETRTVEYQFYN-
LjMLO1 114 GKDKVAFMSQYGIHQLHIFIFVLAIFHILQCITTLALGRTRMAMWKKWEEETKTLEHQFDN-
AtMLO2 150 G--KVAFVSAYGIHQLHIFIFVLAVVHVVYCIVTYAFGKIKMRTWKSWEEETKTIEYQYSN-
AtMLO6 147 G--KVAFVSAYGMHQLHIFIFVLAVCHVIYCIVTYALGKTKMRRWKKWEEETKTIEYQYSH-
AtMLO12 141 G--KVALVSAYGIHQLHIFIFVLAVFHVLYCIITYALGKTKMKKWKSWERETKTIEYQYAN-
StMLO1 111 G--KVQFASSYAIHQLHIFIFVLAVAHVLYCIATFALGRLKMRKWRAWEDETKTIEYQFYN-
SlMLO1 104 G--KVQFASSYAIHQLHIFIFVLAVAHVLYCIATFALGRLKMRKWRAWEDETKTMEYQFYN-
SmMLO1 110 G--KVQFASSYAIHQLHIFIFVLAVAHVLYCIATFALGRLKMRKWRAWEDETKTIEYQFYN-
CaMLO2 110 G--KVQFASSYAIHQLHIFIFVLAIAHVLYCIATFALGRLKMRKWRAWEDETKTIEYQFYN-
NtMLO1 104 G--KVQFASSYAIHQLHIFIFVLAVAHVLYSIATFALGRLKMRKWRAWEEETKTIEYQFYN-
AtMLO7 151 G--SEPLITLKGLHQLHILLFFLAIFHIVYSLITMMLSRLKIRGWKKWEQETLSNDYEFSI-
AtMLO10 149 G--HEPLIPATGLHQLHILLFFMAAFHILYSFITMMLGRLKIRGWKKWEQETCSHDYEFSI-
AtMLO8 167 G--YVELISAEALHQLHILIFFLAIFHVLYSFLTMMLGRLKIRGWKHWENETSSHNYEFST-
AtMLO5 137 G--YVPLISLNALHQVHIFIFFLAVFHVIYSAITMMLGRAKIRGWKVWEEEVI-NDHEMMN-
AtMLO9 144 D--YVALISLNALHQVHIFIFFLAVFHVIYSAITMMLGRAKIRGWKVWEQEVI-HEQEMMN-
AtMLO3 138 G--KTSLISEEGLTQLSYFFFVLACMHILCNLAILLLGMAKMRKWNSWEKETQTVEYLAAN-
AtMLO11 148 G--HEPFVSYEGLEQLHRFIFIMAVTHVTYSCLTMLLAIVKIHSWRIWEDVARLDRHDCLTA
AtMLO14 143 G--HEPFLSYEGMEQLHRFIFIMAVTHVTYSCLTMLLAIVKIHRWRIWEDEVHMDRNDCLTV
AtMLO4 136 G--REPFVSYEGLEQLLRFLFVLGITHVLYSGIAIGLAMSKIYSWRKWEAQAIIMAESDIHA
AtMLO1 146 G--KVPLLSLEALHHLHIFIFVLAISHVTFCVLTVIFGSTRIHQWKKWEDSIADEKFDPETA
AtMLO15 133 G--KVPIMSLSALHELHIFIFVLAVAHIIFCLLTIVFGTMKIKQWKKWEDKVLEKDFDTDQS
AtMLO13 131 G--QVPLVSVEALHQLHIFIFVLAVFHVIFCASTMVLGGARIQQWKHWEDWFKKRPSQKGTT

MtMLO1 202 -DPERF-----RFARDTTFGRRHLSMWTKSPISLWIVCFFRQFFGSISRVDYLALRHGFI
PsMLO1 204 -DPERF-----RFARDTTFGRRHLSMWAQSPILLWIVSFFRQFFGSISRVDYMALRHGFI
LjMLO1 175 -DPERF-----RFARDTTFGRRHLNSWSQSPISLWIVSFFRQFYGSVDKVDYMVLRHGFI
AtMLO2 209 -DPERF-----RFARDTSFGRRHLNFWSKTRVTLWIVCFFRQFFGSVTKVDYLALRHGFI
AtMLO6 206 -DPERF-----RFARDTSFGRRHLSFWSKSTITLWIVCFFRQFFRSVTKVDYLTLRHGFI
AtMLO12 200 -DPERF-----RFARDTSFGRRHLNIWSKSTFTLWITCFFRQFFGSVTKVDYLTLRHGFI
StMLO1 170 -DPERF-----RFARETSFGRRHMHFWSKSPVLLWIVCFFRQFFSSVAKVDYLTLRHGFM
SlMLO1 163 -DPERF-----RFARETSFGRRHLHFWSKSPVLLSIVCFFRQFFSSVAKVDYLTLRHGFM
SmMLO1 169 -DPERF-----RFARETSFGRRHLHFWSKSPLLLWIVCFFRQFFSSVAKVDYLTLRHGFM
CaMLO2 169 -DPERF-----RFARETSFGRRHMHFWSKSPVMLWIVCFFRQFFSSVAKVDYLTLRHGFM
NtMLO1 163 -DPERF-----RFARETSFGRRHLHYWSKSPVLLWIVCFFRQFFSSVAKVDYLTLRHGFM
AtMLO7 210 -DHSRL-----RLTHETSFVREHTSFWTTTPFFFYVGCFFRQFFVSVERTDYLTLRHGFI
AtMLO10 208 -DPSRF-----RLTHETSFVRQHSSFWTKIPFFFYAGCFLQQFFRSVGRTDYLTLRHGFI
AtMLO8 226 -DTSRF-----RLTHETSFVRAHTSFWTRIPFFFYVGCFFRQFFRSVGRTDYLTLRNGFI
AtMLO5 195 -DPSRF-----RLTHETSFVREHVNPWAKNRFSFYVMCFFRQMLRSVRKSDYLTMRHGFI
AtMLO9 202 -DPSRF-----RLTHETSFVREHVNSWASNKFFFYVMCFFRQILRSVRKSDYLTMRHGFI
AtMLO3 197 -DPNRF-----RITRDTTFARRHLSSWTETSFQLWIKCFFRQFYNSVAKVDYLTLRHGFI
AtMLO11 208 VAREKI-----FRRQTTFVQYHTSAPLAKNRILIWVTCFFRQFGRSVDRSDYLTLRKGFI
AtMLO14 203 VAREKI-----FRRQTTFVQYHTSAPLVKNRLLIWVICFFRQFGHSVVRSDYLTLRKGFI
AtMLO4 196 -KKTKV-----MKRQSTFVFHHASHPWSNNRFLIWMLCFLRQFRGSIRKSDYFALRLGFL
AtMLO1 206 LRKRRV----THVHNHAFIKEHFLGIGKDSVILGWTQSFLKQFYDSVTKSDYVTLRLGFI
AtMLO15 193 IKK--F----THVQEHEFIRSRFLGVGKADASLGWVQSFMKQFLASVNESDYITMRLGFV
AtMLO13 191 RRGHHAHAHELFSANHEFFEMHAGGFWRRSVVISWVRSFFKQFYGSVTKSEYIALRQAFI

MtMLO1 256 MAHLAPGNDAEFDFQKYISRSLEKDFKVVVGISPTIWFFAVLFLLTNTHGWYSSYWLPFL
PsMLO1 258 MAHLPPGHDAQFDFQKYISRSIEEDFKVVVGISPTIWLFTVLFLLTNTHGWYSYYWLPFL
LjMLO1 229 IAHLAPGSESKFDFQKYISRSVDEDFKVVVGISPTVWFFAVLILLTNTHGWHSYLWLPFI
AtMLO2 263 MAHFAPGNESRFDFRKYIQRSLEKDFKTVVEISPVIWFVAVLFLLTNSYGLRSYLWLPFI
AtMLO6 260 MAHLAPGSDARFDFRKYIQRSLEEDFKTIVEINPVIWFIAVLFLLTNTNGLNSYLWLPFI
AtMLO12 254 MAHLPAGSAARFDFQKYIERSLEQDFTVVVGISPLIWCIAVLFILTNTHGWDSYLWLPFL
StMLO1 224 MAHLTPQNQNNFDFQIYINRAVDKDFKVVVGISPALWLFTVLYFLTTTDGLYSYLWVPFV
SlMLO1 217 MAHLTPQNQNNFDFQLYINRAVDKDFKVVVGISPALWLFTVLYFLTTTDRLYSYLWVPFI
SmMLO1 223 MAHLTPENQKNFDFQIYINRAVDKDFKVVVGISPALWLFTVLYFLTTTDGLYSYLWVPFV
CaMLO2 223 MAHLTPQNQENFDFQIYINRAVDKDFKVVVGISPALWLFTVLYFLSTTDGVYSYLWVPFV
NtMLO1 217 MAHLTPQNQENFDFQIYINRAVEKDFKFVVEISPALWLFTVLYFLTTTNGLYSYLWVPFI
AtMLO7 264 SAHLAPG--RKFNFQRYIKRSLEDDFKLVVGISPVLWASFVIFLLFNVNGWRTLFWASIP
AtMLO10 262 AAHLAPG--RKFDFQKYIKRSLEDDFKVVVGISPLLWASFVIFLLLNVNGWEALFWASIL
AtMLO8 280 AVHLAPG--SQFNFQKYIKRSLEDDFKVVVGVSPVLWGSFVLFLLLNIDGFKMMFIGTAI
AtMLO5 249 SVHLAPG--MKFNFQKYIKRSLEDDFKVVVGISPELWAFVMLFLLFDVHGWYVTAVITMI
AtMLO9 256 SVHLAPG--MKFDFQKYIKRSLEDDFKVVVGIRPELWAFVMLFLLFDVHGWYVTAVITMI
AtMLO3 251 FAHVSSN--NAFNFQNYIQRSLHEDFKTVVGISPLMWLTVVIFMLLDVSGWRVYFYMSFV
AtMLO11 263 VNHHLT---LKYDFHSYMIRSMEEEFQRIVGVSGPLWGFVVAFMLFNIKGSNLYFWIAII
AtMLO14 258 MNHHLT---LTYDFHSYMIRSMEEEFQKIVGVSGPLWGFVVGFMLFNIKGSNLYFWLAII
AtMLO4 250 TKHNLP---FTYNFHMYMVRTMEDEFHGIVGISWPLWVYAIVCICINVHGLNMYFWISFV
AtMLO1 262 MTHCKGN--PKLNFHKYMMRALEDDFKQVVGISWYLWIFVVIFLLLNVNGWHTYFWIAFI
AtMLO15 247 TTHCKTN--PKFNFHKYLMRALNSDFKKVVGISWYLWVFVVLFLLLNIVAWHVYFWLAFI
AtMLO13 251 MSHCRTN--PSFDFHKYMLRTLEIDFKKVVSISWYLWLFVVVFLLLNVGGWNTYFWLSFL

MtMLO1 316 PLIIILLVGAKLQMIITKMGLRIQDRGEVIKGAPVVEPGDHLFWFNSPNLLLFIIHLVLF
PsMLO1 318 PLIVILLVGAKLQMIITKMGLRIQDRGEVIKGAPVVEPGDHLFWFNRPHLLLFTIHLVLF
LjMLO1 289 PLIIILLVGTKLQMIITNMGLKIQERGDVIKGAPLVEPGDDLFWFNRPRLILSLVHLVLF
AtMLO2 323 PLVVILIVGTKLEVIITKLGLRIQEKGDVVRGAPVVQPGDDLFWFGKPRFILFLIHLVLF
AtMLO6 320 PFIVILIVGTKLQVIITKLGLRIQEKGDVVKGTPLVQPGDHFFWFGRPRFILFLIHLVLF
AtMLO12 314 PLIVILIVGAKLQMIISKLGLRIQEKGDVVKGAPVVEPGDDLFWFGRPRFILFLIHLVLF
StMLO1 284 PLVIILLVGTKLQMIITEMGVRISERGDIVKGVPVVETGDHLFWFNRPGLVLFLINFVLF
SlMLO1 277 PLVIILLVGTKLQMIITEMGVRISERGDIVKGVPVVETGDHLFWFNRPALVLFLINFVLF
SmMLO1 283 PLIIILLVGTKLQMIITEMGVRISERGDIVKGVPVVETGDHLFWFNRPGLVLFLINFVLF
CaMLO2 283 PLIIILLVGTKLQMIITEMGVRISERGDIVKGVPVVEIGDHLFWFNRPGLVLFFINFVLF
NtMLO1 277 PLVIILLVGTKLEMIIAEMGVRISKRGDIVRGVPVVETGDHLFWFNRPGFVLFLINFVLF
AtMLO7 322 PLLIILAVGTKLQAIMATMALEIVETHAVVQGMPLVQGSDRYFWFDCPQLLLHLIHFALF
AtMLO10 320 PVLIILAVSTKLQAILTRMALGITERHAVVQGIPLVHGSDKYFWFNRPQLLLHLLHFALF
AtMLO8 338 PVIIILAVGTKLQAIMTRMALGITDRHAVVQGMPLVQGNDEYFWFGRPHLILHLMHFALF
AtMLO5 307 PPLLTLAIGTKLQAIISDMALEIQERHAVIQGMPLVNVSDRHFWFSRPALVLHIIHFILF
AtMLO9 314 PPLLTLAIGTKLQAIISYMALEIQERHAVIQGMPVVNVSDQHFWFEKPDLVLHMIHFVLF
AtMLO3 309 PLIIVLVIGTKLEMIVAKMAVTIKENNSVIRGTPLVESNDTHFWFSNPRFLLSILHYTLF
AtMLO11 320 PVTLVLLVGAKLQHVIATLALE-NAGLTEYPSGVKLRPRDELFWFNKPELLLSLIHFILF
AtMLO14 315 PITLVLLVGAKLQHVIATLALE-NASITEYASGIKLRPRDELFWFKKPELLLSLIHFIQF
AtMLO4 307 PAILVMLVGTKLEHVVSKLALEVKEQQTGTSNGAQVKPRDGLFWFGKPEILLRLIQFIIF
AtMLO1 320 PFALLLAVGTKLEHVIAQLAHEVAEKHVAIEGDLVVKPSDEHFWFSKPQIVLYLIHFILF
AtMLO15 305 PLILLLAVGTKLEHIITDLAHEVAEKHIAVEGDLVVRPSDDLFWFQSPRLVLFLIHFILF
AtMLO13 309 PLILLLMVGAKLEYIISSLALDVSEKRSRAE-EAVITPSDELFWFHRPGIVLQLIHFILF

MtMLO1 376 QNAFQLAFFSWSTYE--FSINSCFHRTTADNVIRVSVGILIQFLCSYVTLPLYALVTQMG
PsMLO1 378 QNAFQLAFFAWSTYE--FSITSCFHKTTADSVIRITVGVVIQTLCSYVTLPLYALVTQMG
LjMLO1 349 QNAFQLAFFAWSACDNDFKINSCFHRSTADVVIRLTLGVVTQVLCSYVTLPLYALVTQMG
AtMLO2 383 TNAFQLAFFAWSTYE--FNLNNCFHESTADVVIRLVVGAVVQILCSYVTLPLYALVTQMG
AtMLO6 380 TNAFQLAFFVWSTYE--FGLKNCFHESRVDVIIRISIGLLVQILCSYVTLPLYALVTQMG
AtMLO12 374 TNAFQLAFFVWSTYE--FTLKNCFHHKTEDIAIRITMGVLIQVLCSYITLPLYALVTQMG
StMLO1 344 QNAFQVAFFVWSWWK--FGFPSCFHQNAADLAIRLTMGVIIQVHCSYVTLPLYALVTQMG
SlMLO1 337 QNAFQVAFFFWSWWK--FGFPSCFHKNAADLAIRLTMGVIIQVHCSYVTLPLYALVTQMG
SmMLO1 343 QNAFQVAFFVWSWWK--FDFPSCFHKNAADLAIRLTMGVIIQVHCSYVTLPLYALVTQMG
CaMLO2 343 QNAFQVAFFVWSWWK--FGFPSCFHRNAADLAIRLTMGVIIQVHCSYVTLPLYALVTQMG
NtMLO1 337 QNAFQVAFFVWSWWK--FSYPSCFHQNAADIAIRLTMGVIIQVHCSYVTLPLYALVTQMG
AtMLO7 382 QNAFQITHFFWIWYS--FGLKSCFHKDFNLVVSKLFLCLGALILCSYITLPLYALVTQMG
AtMLO10 380 QNAFQLTYFFWVWYS--FGLKSCFHTDFKLVIVKLSLGVGALILCSYITLPLYALVTQMG
AtMLO8 398 QNAFQITYFFWIWYS--FGSDSCYHPNFKIALVKVAIALGVLCLCSYITLPLYALVTQMG
AtMLO5 367 QNAFEITYFFWIWYE--FGLRSCFHHHFALIIIRVALGVGVQFLCSYITLPLYALVTQMG
AtMLO9 374 QNAFEITYFFWIWYE--FGLRSCFHHHFGLIIIRVCLGVGVQFLCSYITLPLYALVTQMG
AtMLO3 369 LNTFEMAFIVWITWQ--FGINSCYHDNQGIIITRLVLAVTVQFLSSYITLPLYAIVTQMG
AtMLO11 379 QNSFELASFFWFWWQ--FGYSSCFLKNHYLVYFRLLLGFAGQFLCSYSTLPLYALVTQMG
AtMLO14 374 QNAFELASFFWFWWQ--FGYNSCFLRNHLLVYLRLILGFSGQFLCSYSTLPLYALVTQMG
AtMLO4 367 QNAFEMATFIWFLWG--IKERSCFMKNHVMISSRLISGVLVQFWCSYGTVPLNVIVTQMG
AtMLO1 380 QNAFEIAFFFWIWVT--YGFDSCIMGQVRYIVPRLVIGVFIQVLCSYSTLPLYAIVSQMG
AtMLO15 365 QNSFEIAYFFFILFQ--FGWDSCIMDHVKFVIPRLVIGVIIQLLCSYSTLPLYALVTQMG
AtMLO13 368 QNSFEIAFFFWILFT--YGIHSCIMEKLGYLIPRLVMGVLVQVLCSYSTLPLYALVTQMG

MtMLO1 434 STMKPTIFNERLATALKKWHHTAKKQVKHNKHSNN--TTPYSSRQSTPTHGMSPVHLLHR
PsMLO1 436 STMKPTIFNERVATALKNWHHTAKKQVKQSNHSNN--TTPYSSRPSTPTHAMSPVHLLHR
LjMLO1 409 STMRPTIFHDRVATALKSWHHTAKKHVKHNRDSNSHSNTPFSSRPATPTHGMSPVHLLHK
AtMLO2 441 SKMKPTVFNDRVATALKKWHHTAKNETKHGRHSGS--NTPFSSRPTTPTHGSSPIHLLHN
AtMLO6 438 SKMKPTVFNERVATALKSWHHTAKKNIKHGRTSES--TTPFSSRPTTPTHGSSPIHLLRN
AtMLO12 432 TSMRPTIFNDRVANALKKWHHTAKKQTKHG-HSGS--NTPHSSRPTTPTHGMSPVHLLHN
StMLO1 402 SSMKPIIFGDNVATALRSWHHTAKKRVKHG-LSGH--TTPANSRPTTPLHGTSPVHLLRG
SlMLO1 395 SSMKPIIFGDNVATALRSWHHTAKKRVKHG-LSGH--TTPANSRPTTPLRGTSPVHLLRG
SmMLO1 401 SSMKPIIFGDNVATALRSWHHMAKKRVKHGRLSGN--TTPVSSRPTTPLHGTSPVHLLRG
CaMLO2 401 SSMKPIIFGDNVATALRSWHNTAKKRVRHGRVSEN--TTPISSRPATPLRGTSPVHLLRG
NtMLO1 395 TSMKPIIFGDNVATALRSWHNTAKKRVKHGRLSEN--TTPVSSRPATPLHGTSPVHLLRS
AtMLO7 440 SHMKKAVFDEQMAKALKKWHKDIKLKKGKARKLPSKTLGVSESFSLSSSSSATTLHRSKT
AtMLO10 438 SNMKKAVFDEQMAKALKKWHMTVKKKKGKARKPPTETLGVSDTVSTSTSSFHASGATLLR
AtMLO8 456 SRMKKSVFDEQTSKALKKWRMAVKKKKGVKATTKRLGGDGSASPTASTVRSTSSVRSLQR
AtMLO5 425 STMKRSVFDDQTSKALKNWHKNAKKKSETPGQTQPPLPNLRPKTGGDIESASPANITASV
AtMLO9 432 STMKRSVFDEQTSKALEQWHKKARKKNEK-------------------------------
AtMLO3 427 SSYKRAILEEQLANVLRHWQGMVRDKKKTIQTPDTDNNSNNNNGDIDSGESPVQTEVASE
AtMLO11 437 TNYKAALIPQRIRETIRGWGKATRRKRRHGLYGDDSTVRTETSTIASLEEYDHQVLDVTE
AtMLO14 432 TNYKAALLPQRVRETINGWGKATRRKRRHGLYGDDSTIRTETSTIASVDEYNDQVLDVSE
AtMLO4 425 SRHKKAVIAESVRDSLHSWCKRVKERSKHTRSVCSLDTATIDERDEMTVGTLSRSSSMTS
AtMLO1 438 SSFKKAIFEENVQVGLVGWAQKVKQKRDLKAAASNGDEGSSQAGPGPDSGSGSAPAAGPG
AtMLO15 423 SSFKGAIFNEQTQEHLVGWAKMAKRGVKKGATQVGTSHDATSPRPSIQLNSLLGKGSSQQ
AtMLO13 426 SKFKKGIFDNVVQSTLEGWLEDTRNRGESTSEAHRIEMQPTTPESYNVQSENP-------

MtMLO1 492 QTFGNSDSLQTSPRTSNYENEQWDVEGGGSTSPRNNQTVASEIEIPIVESFSTTELPVSV
PsMLO1 494 HTAGNSDSLQTSPEKSDYKNEQWDIEGEGPTSLRNDQTGQHEIQIAGVESFSSTELPVRI
LjMLO1 469 HHNYHNSDSPLASPRESPSNYETEQWYLEPNSPSNHTRGHDQTLQMQVLGSSATEFSPAE
AtMLO2 499 FNNRSVENYPSSPSPRYSGHGHHEHQFWDPESQHQEAETSTHHSLAHESSEPVLASVELP
AtMLO6 496 APHKRSRSVDESFANSFSPRNSDFDSWDPESQHETAETSNSNHRSRFGEEESEKKFVSSS
AtMLO12 489 YNNRSLDQQTSFTASPSPPRFSDYSGQGHGHQHFFDPESQNHSYQREITDSEFSNSHHPQ
StMLO1 459 YPQYNEDSVQASPRTSNVENEGWANEISNDNQEGEILQHASTDHNKQIEITMSDFTFGNK
SlMLO1 452 YPQYNEDSVQASPRTSNVENEGWANE----NQEGEILQHASTDHNKQIEITMSDFTFGNK
SmMLO1 459 YPQYNEDSVQASPRTSNVENEGWANEISTDNKDYQEGHASTSVRPPHAHNQQIEITMSDF
CaMLO2 459 YPKYNEDNVQAYPRTSNVENEGWANETSTENKDHQEEGQILQHASTSMQHPHTDQHQIEI
NtMLO1 453 YPQYSNEESRTSNAENEGWANEIPTSPRRQIENIKDDDHQEGEIHASSSVHQVEIAMSEF
AtMLO7 500 TGHSSNIIYYKQEDEEDEMSDLEAGAEDAIDRIQQQEMQFHNS-----------------
AtMLO10 498 SKTTGHSTASYMSNFEDQSMSDLEAEPLSPEPIEGHTLVRVGDQNTEIEYTGDISPGNQF
AtMLO8 516 YKTTPHSMRYEGLDPETSDLDTDNEALTPPKSPPSFELVVKVEPNKTNTGETSRDTETDS
AtMLO5 485 DVKESDQSQSRDLLSGP-------------------------------------------
AtMLO9 ------------------------------------------------------------
AtMLO3 487 FRFSGRQSPILQEIQIQEKTER--------------------------------------
AtMLO11 497 TSFEQQRKQQEQGTTELELQPIQPRNDCVPNDTSSRVGTPLLRPWLSISSPTTTIELRSE
AtMLO14 492 TSPVQDNELELQLIRGACGNSSSVETPILRPCASISSTTFSRLQTETTDSLSRSSSLPMR
AtMLO4 485 LNQITINSIDQAESIFGAAASSSSPQDGYTSRVEEYLSETYNNIGSIPPLNDEIEIEIEG
AtMLO1 498 AGFAGIQLSRVTRNNAGDTNNEITPDHNN-------------------------------
AtMLO15 483 NQNPKEKSEIAHHD----------------------------------------------
AtMLO13 ------------------------------------------------------------

MtMLO1 552 RHEIGTTSSSKDFSFEKRHIGSN------
PsMLO1 554 RHESTSGSKDFSFEKRHLGSN--------
LjMLO1 529 VHHEITPIGLPEFSFDKAPTSRE------
AtMLO2 559 PIRTSKSLRDFSFKK--------------
AtMLO6 556 VELPPGPGQIRTQHEISTISLRDFSFKR-
AtMLO12 549 VDMASPVREEKEIVEHVKVDLSEFTFKK-
StMLO1 519 X----------------------------
SlMLO1 508 X----------------------------
SmMLO1 519 TFGNKX-----------------------
CaMLO2 519 AMSDFTFGNKX------------------
NtMLO1 513 TFGNKMS----------------------
AtMLO7 -----------------------------
AtMLO10 558 SFVKNVPANDID-----------------
AtMLO8 576 KEFSFVKPAPSNESSQDR-----------
AtMLO5 -----------------------------
AtMLO9 -----------------------------
AtMLO3 -----------------------------
AtMLO11 557 PMETLSRSSSLPSEKRV------------
AtMLO14 552 REC--------------------------
AtMLO4 545 EEDNGGRGSGSDENNGDAGETLLELFRRT
AtMLO1 -----------------------------
AtMLO15 -----------------------------
AtMLO13 -----------------------------

CaMBD

**Supplementary Fig. 2** Nucleotide multiple alignment of full-length coding sequences of eggplant *SmMLO1*, potato *StMLO1*, tobacco *NtMLO1*, tomato *SlMLO1* and pepper *CaMLO2*.

StMLO1 1 ATGGCTAAAGAACGGTCGATGGAGGCAACCCCTACTTGGGCGATTGCTGTGGTTTGCTTC
SlMLO1 1 ATGGCTAAAGAACGGTCTATGGAGGCAACCCCTACGTGGGCAATTGCTGTGGTTTGCTTC
SmMLO1 1 ATGGCTAAAGAACGGTCGATGGAGGGAACCCCCACTTGGGCGGTTGCCGTCGTTTGCTTC
NtMLO1 1 ATGGCTAAAGAACGGTCGATGGAGGCAACTCCGACTTGGGCAGTTGCCGCAGTTTGCTTC
CaMlo2 1 ATGGCTAAAGAACGGTCGATGGAGGCAACCCCTACGTGGGCGGTTGCCGTGGTTTGCTTC


StMLO1 61 ATCTTGCTCGCTATTTCCATTTTTATTGAACAAATTATTCATCATATTGGAGAGTGGTTA
SlMLO1 61 ATCTTGCTCGCTATTTCTATTTTTATTGAACAAATTATTCATCACATTGGAGAGTGGTTA
SmMLO1 61 ATCTTGCTGGCTATTTCCATTTTTATTGAACAAATTATTCATCACCTTGGAGAGTGGTTA
NtMLO1 61 ATCTTGCTGGCTATTTCCATTTTCATTGAACAAATTATTCATCATCTTGGAGAGTGGTTG
CaMlo2 61 ATCTTGCTGGCTATTTCCATTTKTATTGAACAAATTATGCATCACCTTGGAGAGTGGTTG


StMLO1 121 CTGGAAAAGCGGAAAAAGCCTCTATATGAAGCACTTGAAAAGATCAAAGCTGAACTTATG
SlMLO1 121 CTGGAAAAGCGGAAAAAGTCTCTATATGAAGCACTTGAAAAGATCAAAGCTGAACTTATG
SmMLO1 121 TTGGAAAAGCACAAAAAGCCACTACACGAAGCACTTGAGAAGATCAAAGCAGAACTTATG
NtMLO1 121 TTGAAAAAACATAAAAAGCCTCTTTATGAAGCACTTGAAAAGATCAAAGCAGAACTGATG
CaMlo2 121 TTGAAAAAACACAAAAAGCCTCTATACGAAGCACTTGAAAAGATCAAAGCAGAGCTTATG


StMLO1 181 CTGTTGGGATTCTTATCACTGTTGTTGACAGTGTTGCAAGAACCAGTTTCTAACTTATGC
SlMLO1 181 CTGTTGGGATTCTTATCACTGTTGTTGACAGTGTTGCAAGATCCAGTTTCTAACTTATGT
SmMLO1 181 CTGTTGGGATTCATATCACTGCTGTTGACAGTGGTGCAAGATCCAGTTTCTAACATATGC
NtMLO1 181 TTGTTGGGATTCATATCACTGCTGTTGACAGTGGTGCAAAGCCCAGTGTCTAACTTATGC
CaMlo2 181 TTGTTGGGATTCATATCATTGTTGTTGACAGTGATACAAGACCCAGTTTCTAACTTATGT


StMLO1 241 GTCCCCAAGAGTATTGGTTATTCATGGCATCCTTGTAAGCCAAAGGCAGACGCCCAGTCT
SlMLO1 241 GTCCCCAAGAGTGTTGGTTATTCATGGCATCCTTGTATGGCAAAGGAAGATGCCAAGTCT
SmMLO1 241 GTGCCCAAAACTGTTGGTTATTCGTGGCATCCTTGTAAGGCACAGGAAGACGACAAGCCT
NtMLO1 241 GTGCCAAAGAGTGTTGGTTATTCTTGGCATCCTTGTAAGTCTGATGAAGCTGCCAAGAAT
CaMlo2 241 GTCCCCAAAAGTGTTGGTTATTCCTGGCATCCTTGTAAGGCAGATGAAGATGTCAAGTCT


StMLO1 301 GAGTATGAGGTTACTTCATGCGACAAAAAGGGAAAAGTCCAATTTGCATCTTCATATGCA
SlMLO1 301 GAGTATGATGACCCTT---GTCTACCAAAGGGAAAAGTGCAATTTGCATCTTCATATGCA
SmMLO1 301 AAGTATGATGACCCTT---GTCTAGAAAAGGGAAAAGTCCAATTTGCTTCTTCATATGCA
NtMLO1 301 AAATATGATGACCCTT---GTCTACCAAAGGGAAAAGTCCAATTTGCATCTTCATATGCA
CaMlo2 301 GAGTATGATGACCCTT---GTTTACAAAAGGGAAAAGTTCAATTTGCATCTTCATATGCA


StMLO1 361 ATACACCAGCTCCATATCTTCATCTTTGTGTTGGCAGTTGCTCATGTATTGTACTGTATA
SlMLO1 358 ATACACCAGCTCCATATCTTCATCTTTGTATTGGCAGTTGCTCATGTATTGTACTGTATA
SmMLO1 358 ATACACCAGCTCCATATCTTCATCTTTGTGTTGGCAGTTGCTCATGTATTGTATTGTATA
NtMLO1 358 ATACACCAGCTCCACATTTTCATCTTTGTCTTGGCAGTTGCTCATGTATTATACTCTATA
CaMlo2 358 ATACACCAGCTCCATATCTTCATCTTTGTGTTGGCAATTGCGCATGTTTTGTACTGTATA


StMLO1 421 GCAACTTTTGCTTTGGGCAGACTAAAGATGAGAAAATGGAGGGCATGGGAGGATGAAACA
SlMLO1 418 GCAACTTTTGCTTTGGGCAGGCTAAAGATGAGAAAATGGAGGGCATGGGAGGATGAAACA
SmMLO1 418 GCAACTTTTGCTTTGGGCAGGCTAAAGATGAGAAAATGGAGGGCCTGGGAAGATGAAACT
NtMLO1 418 GCAACTTTTGCTTTAGGCAGGCTAAAGATGAGAAAATGGAGAGCCTGGGAGGAAGAAACA
CaMlo2 418 GCAACTTTTGCTTTGGGCAGGTTAAAGATGAGAAAATGGAGGGCCTGGGAGGATGAAACA

StMLO1 481 AAAACAATTGAGTACCAATTCTACAACGACCCTGAGAGATTCAGATTTGCAAGGGAGACC
SlMLO1 478 AAAACAATGGAGTACCAATTCTACAACGACCCTGAGAGATTCAGATTTGCAAGGGAGACC
SmMLO1 478 AAAACAATTGAATACCAATTCTACAACGATCCTGAGAGATTCAGATTTGCAAGGGAGACC
NtMLO1 478 AAAACAATTGAGTACCAATTCTACAACGATCCAGAGAGGTTCAGATTTGCAAGGGAGACG
CaMlo2 478 AAAACAATTGAGTACCAATTCTATAACGAYCCTGAGAGGTTTAGGTTTGCAAGGGAGACC


StMLO1 541 TCGTTTGGACGTAGGCATATGCATTTCTGGAGCAAGTCGCCCGTGTTGCTCTGGATAGTT
SlMLO1 538 TCGTTTGGACGTAGGCATTTGCATTTCTGGAGCAAGTCCCCCGTGTTGCTCTCGATAGTT
SmMLO1 538 TCCTTTGGACGTAGGCATTTGCATTTCTGGAGCAAGTCACCGCTGTTGCTCTGGATAGTT
NtMLO1 538 TCATTTGGACGTAGGCACTTGCATTATTGGAGCAAGTCTCCAGTGCTGCTCTGGATAGTT
CaMlo2 538 TCATTTGGACGTAGGCATATGCATTTTTGGAGCAAGTCGCCGGTGATGCTCTGGATAGTT

StMLO1 601 TGTTTCTTCAGGCAATTCTTCTCATCAGTAGCAAAAGTTGACTATTTAACCCTTAGACAT
SlMLO1 598 TGTTTCTTTCGGCAATTCTTCTCATCAGTTGCAAAAGTTGACTATTTAACCCTTAGACAT
SmMLO1 598 TGTTTCTTCAGGCAATTCTTCTCCTCAGTAGCAAAGGTTGACTATTTAACCCTTAGACAT
NtMLO1 598 TGTTTCTTCAGGCAATTCTTCTCATCAGTAGCAAAAGTTGACTATCTAACCCTTAGACAT
CaMlo2 598 TGTTTCTTCAGGCAATTCTTTTCATCAGTAGCAAAAGTTGACTATTTAACCCTTAGACAT


StMLO1 661 GGGTTCATGATGGCACATTTAACTCCACAAAATCAAAATAATTTTGATTTTCAAATATAC
SlMLO1 658 GGGTTCATGATGGCACATTTAACTCCACAAAATCAAAATAATTTTGATTTTCAATTATAC
SmMLO1 658 GGGTTCATGATGGCACATTTAACTCCAGAGAATCAAAAGAATTTTGATTTTCAAATATAC
NtMLO1 658 GGGTTCATGATGGCACATTTAACTCCACAGAATCAGGAAAATTTTGATTTCCAGATATAC
CaMlo2 658 GGGTTCATGATGGCACATTTAACACCACAGAATCAAGAGAACTTTGATTTTCAAATATAC


StMLO1 721 ATTAACAGAGCAGTTGACAAAGACTTCAAAGTTGTTGTTGGAATAAGTCCTGCATTATGG
SlMLO1 718 ATTAACAGAGCAGTTGACAAAGACTTCAAAGTTGTTGTTGGAATAAGTCCTGCATTATGG
SmMLO1 718 ATTAACAGAGCAGTTGACAAAGACTTCAAAGTTGTTGTGGGAATAAGTCCAGCATTATGG
NtMLO1 718 ATCAATAGAGCAGTTGAAAAAGACTTCAAATTTGTTGTGGAAATAAGTCCAGCATTATGG
CaMlo2 718 ATTAATAGAGCAGTTGACAAAGATTTCAAAGTTGTCGTGGGAATAAGTCCAGCATTATGG


StMLO1 781 CTCTTCACGGTGCTATATTTTCTGACTACTACCGATGGATTGTACTCGTATCTTTGGGTG
SlMLO1 778 CTCTTCACGGTGCTATATTTTCTGACTACTACCGATCGATTGTACTCGTATCTTTGGGTG
SmMLO1 778 CTCTTCACGGTACTATATTTTCTAACGACTACCGATGGACTATACTCGTACCTTTGGGTG
NtMLO1 778 CTCTTCACAGTACTATATTTTCTAACCACTACCAATGGATTGTACTCGTACCTTTGGGTG
CaMlo2 778 CTCTTCACGGTATTATATTTTCTATCCACCACCGATGGAGTTTACTCGTATCTTTGGGTT


StMLO1 841 CCATTTGTCCCACTTGTAATAATATTGCTGGTTGGCACAAAACTTCAAATGATCATAACA
SlMLO1 838 CCATTTATCCCACTTGTAATAATATTGCTAGTTGGCACAAAACTTCAAATGATCATAACA
SmMLO1 838 CCATTTGTCCCACTCATAATAATATTGCTGGTTGGCACAAAACTTCAAATGATCATAACA
NtMLO1 838 CCATTTATCCCGTTAGTAATAATATTGCTGGTTGGCACAAAACTTGAAATGATAATAGCA
CaMlo2 838 CCATTTGTCCCACTCATTATAATATTGTTGGTTGGGACAAAACTTCAAATGATCATAACA


StMLO1 901 GAAATGGGAGTAAGGATTTCAGAAAGGGGAGACATAGTAAAAGGTGTACCAGTGGTGGAG
SlMLO1 898 GAAATGGGAGTAAGGATTTCAGAAAGGGGAGACATAGTAAAAGGTGTACCTGTGGTGGAG
SmMLO1 898 GAAATGGGGGTAAGGATTTCAGAAAGGGGAGACATAGTGAAAGGTGTGCCAGTGGTGGAG
NtMLO1 898 GAAATGGGAGTAAGGAATTCCAAGAGGGGAGACATAGTGAGAGGTGTACCAGTGGTGGAG
CaMlo2 898 GAAATGGGGGTTAGAATTTCAGAAAGGGGAGACATAGTGAAAGGTGTACCAGTGGTGGAG


StMLO1 961 ACTGGAGACCATCTTTTCTGGTTTAATCGCCCTGGCCTTGTGTTATTCTTGATTAACTTT
SlMLO1 958 ACTGGTGACCATCTTTTCTGGTTTAATCGCCCTGCCCTTGTCCTATTCTTGATTAACTTT
SmMLO1 958 ACTGGTGACCATCTTTTTTGGTTTAATCGCCCTGGCCTTGTGCTTTTCTTGATTAATTTT
NtMLO1 958 ACAGGTGACCATCTTTTCTGGTTCAACCGACCTGGCTTTGTCCTTTTCTTGATTAACTTT
CaMlo2 958 ATCGGTGACCATCTTTTCTGGTTTAATCGCCCTGGCCTTGTGCTTTTCTTCATTAACTTT


StMLO1 1021 GTGCTCTTTCAGAATGCGTTTCAAGTTGCTTTCTTTGTTTGGAGTTGGTGGAAATTTGGT
SlMLO1 1018 GTACTCTTTCAGAATGCGTTTCAAGTTGCTTTCTTTTTTTGGAGTTGGTGGAAATTTGGT
SmMLO1 1018 GTGCTCTTTCAGAATGCGTTTCAAGTCGCTTTCTTTGTTTGGAGTTGGTGGAAATTTGAC
NtMLO1 1018 GTGCTCTTTCAGAATGCATTCCAAGTTGCTTTCTTCGTTTGGAGTTGGTGGAAATTTAGT
CaMlo2 1018 GTCCTCTTTCAGAATGCGTTTCAAGTTGCTTTCTTTGTTTGGAGTTGGTGGAAATTTGGT


StMLO1 1081 TTTCCATCTTGCCTTCATCAGAATGCTGCAGACCTAGCCATAAGGCTAACCATGGGGGTG
SlMLO1 1078 TTCCCATCTTGCTTTCATAAGAATGCTGCAGACCTAGCCATAAGGCTAACCATGGGGGTG
SmMLO1 1078 TTTCCGTCTTGCTTTCACAAGAATGCTGCAGACCTAGCCATAAGGCTAACCATGGGGGTG
NtMLO1 1078 TACCCATCTTGCTTCCACCAGAATGCTGCAGATATAGCCATAAGGCTGACCATGGGGGTG
CaMlo2 1078 TTTCCATCCTGCTTTCATAGAAATGCTGCAGACCTAGCCATTAGGCTAACCATGGGAGTA


StMLO1 1141 ATCATACAGGTCCATTGCAGCTATGTGACTCTCCCTCTTTATGCCTTAGTTACCCAGATG
SlMLO1 1138 ATCATACAGGTCCATTGCAGCTATGTGACTCTCCCTCTTTATGCCTTAGTTACACAGATG
SmMLO1 1138 ATCATACAGGTCCATTGCAGCTATGTGACTCTTCCTCTCTATGCCTTAGTCACCCAGATG
NtMLO1 1138 ATCATACAGGTCCATTGCAGCTATGTGACTCTCCCTCTTTATGCCTTGGTCACACAGATG
CaMlo2 1138 ATCATACAAGTCCATTGCAGCTATGTAACTCTCCCTCTATATGCCTTAGTTACTCAGATG

StMLO1 1201 GGTTCATCAATGAAGCCTATCATCTTTGGTGATAATGTGGCAACAGCTCTTAGAAGCTGG
SlMLO1 1198 GGTTCATCAATGAAGCCTATCATCTTTGGTGATAATGTGGCAACAGCTCTTAGAAGCTGG
SmMLO1 1198 GGTTCATCAATGAAACCTATCATCTTTGGTGATAATGTGGCAACAGCTCTTAGAAGCTGG
NtMLO1 1198 GGAACATCAATGAAACCTATAATCTTTGGTGATAATGTGGCAACAGCTCTTAGAAGCTGG
CaMlo2 1198 GGTTCATCAATGAAGCCTATCATCTTTGGTGATAATGTGGCAACAGCTCTTAGAAGCTGG


StMLO1 1261 CACCATACAGCGAAAAAGCGGGTGAAACATGGGC---TATCAGGACACACCACTCCTGCC
SlMLO1 1258 CACCATACAGCGAAAAAACGGGTGAAACATGGGC---TATCAGGACATACCACCCCTGCA
SmMLO1 1258 CACCATATGGCGAAAAAGCGAGTGAAACATGGGCGGCTATCGGGAAACACCACCCCTGTC
NtMLO1 1258 CACAACACGGCGAAAAAGCGGGTGAAACACGGCCGGCTATCGGAAAACACCACCCCTGTC
CaMlo2 1258 CACAATACAGCGAAAAAGCGGGTGAGACATGGGCGGGTATCAGAAAACACCACTCCGATA


StMLO1 1318 AACAGCAGACCAACGACACCATTGCATGGTACCTCCCCTGTTCACTTATTACGTGGTTAT
SlMLO1 1315 AACAGCAGACCAACCACACCATTGCGTGGTACCTCCCCTGTTCACTTATTACGCGGTTAT
SmMLO1 1318 TCCAGCAGACCGACCACACCTTTGCATGGTACTTCCCCGGTTCACTTATTGCGCGGTTAC
NtMLO1 1318 TCTAGCAGACCGGCCACACCGTTGCATGGTACCTCGCCGGTTCACTTATTACGCAGTTAC
CaMlo2 1318 TCTAGCAGACCGGCCACACCATTGCGTGGTACCTCCCCAGTTCACTTGCTACGTGGCTAC


StMLO1 1378 CCACAATATAATGAGGATAGTGTTCAAGCATCTCCTCGGACATCCAACGTTGAAAATGAA
SlMLO1 1375 CCACAATATAATGAGGACAGTGTTCAAGCATCTCCTCGGACATCCAATGTCGAAAATGAA
SmMLO1 1378 CCACAATACAATGAGGACAGTGTTCAAGCATCTCCTCGGACATCCAACGTCGAAAATGAA
NtMLO1 1378 CCACAATATAGTAATGAGGAGAGTCGGACATCCAATGCGGAAAATGAAGGCTGGGCTAAT
CaMlo2 1378 CCAAAATATAACGAGGACAATGTTCAAGCATATCCTCGAACATCGAATGTAGAAAATGAA


StMLO1 1438 GGCTGGGCTAATGAAATATCCAATGACAATCAGGAGGGAGAGATCCTGCAGCATGCCTCC
SlMLO1 1435 GGGTGGGCTAATGAAAATCAGGAGGGAGAGATCCTGCAGCATGCCTCCACTGATCATAAC
SmMLO1 1438 GGGTGGGCTAATGAAATATCTACTGACAATAAAGATTATCAGGAGGGACATGCCTCCACA
NtMLO1 1438 GAAATACCAACCTCTCCTCGTAGACAAATTGAGAATATTAAAGATGATGATCATCAGGAG
CaMlo2 1438 GGCTGGGCTAATGAAACATCCACTGAGAATAAAGATCATCAGGAGGAGGGACAAATCCTG


StMLO1 1498 ACTGATCATAACAACCAAATTGAGATTACAATGTCAGATTTCACTTTTGGAAACAAATAA
SlMLO1 1495 AAGCAAATTGAGATTACAATGTCAGATTTTACTTTTGGAAACAAATAAATGTAAAAACGA
SmMLO1 1498 TCTGTGCGACCTCCCCATGCTCACAACCAGCAAATTGAGATTACAATGTCAGATTTTACT
NtMLO1 1498 GGAGAAATCCATGCCTCCAGCTCTGTGCATCAAGTTGAGATTGCAATGTCAGAATTCACA
CaMlo2 1498 CAGCATGCCTCCACTTCTATGCAACATCCGCATACTGATCAACATCAAATTGAGATTGCA

StMLO1 1558 AAGATCAATGTATATGTAAAAACTAATTTCCTCTGCGTTGTTTAAGTTCATTACTGTAGA
SlMLO1 1555 ATTTTCTTCTTCATTGTTTTAAGTTCATTACTGTAGTTCAAATGGCAATGATTTTGTAAA
SmMLO1 1558 TTTGGAAACAAATAA---------------------------------------------
NtMLO1 1558 TTTGGCAACAAAATGAGTTGA---------------------------------------
CaMlo2 1558 ATGTCAGATTTTACTTTTGGAAACAAATAG------------------------------


StMLO1 1618 GAGCATAAATGGATCCTAGATAGTTCAAGTGCCTGCTTGCTAATACACAAAGATTTTCAT
SlMLO1 1615 ATTTTATACAGAGGTACTCATGCATGGTGCTCTTCATTTCAAGGTAAGAACCTTCTTATA
SmMLO1 ------------------------------------------------------------
NtMLO1 ------------------------------------------------------------
CaMlo2 ------------------------------------------------------------


StMLO1 1678 TCCAAAAAAAAAAAAAAA--------
SlMLO1 1675 TCGATTTATAGCTACTTTACATCTCA
SmMLO1 --------------------------
NtMLO1 --------------------------
CaMlo2 --------------------------

**Supplementary Fig. 3** Segregation of the T_2__a (left) and T_2__b (right) families for markers derived from the nptII gene (panels A and C) and 35S promoter (panels B and D), indicating the presence of the 35S::*NtMLO1* construct.


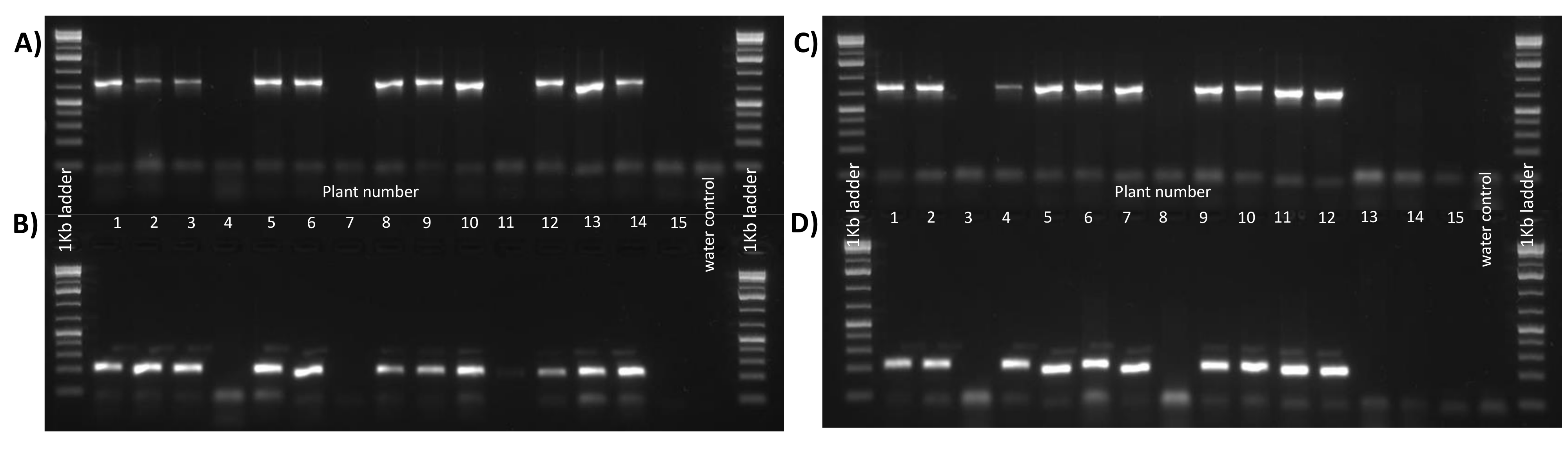


**Supplementary Fig. 4** Relative quantification of *NtMLO*1 expression levels in complementation tests, assessed by qPCR. Data refer to 11 and 10 individuals of two T_2_ families [T_2_(+)_a and T_2_(+)_b] positive for the presence of the overexpression construct harbouring wild-type *NtMLO1*; 11 and 7 individuals of two T_2_ families [T_2_(+)_Q198R-a and b] positive for the presence of the overexpression construct harbouring a *NtMLO1* mutant sequence, resulting in the substitution of a glutamine residue with arginine; 18 non-transgenic individuals from the 4 T_2_ families above mentioned [T_2_(-)]; 10 individuals of the Slmlo1 mutant line, used as background genotype for transformation.


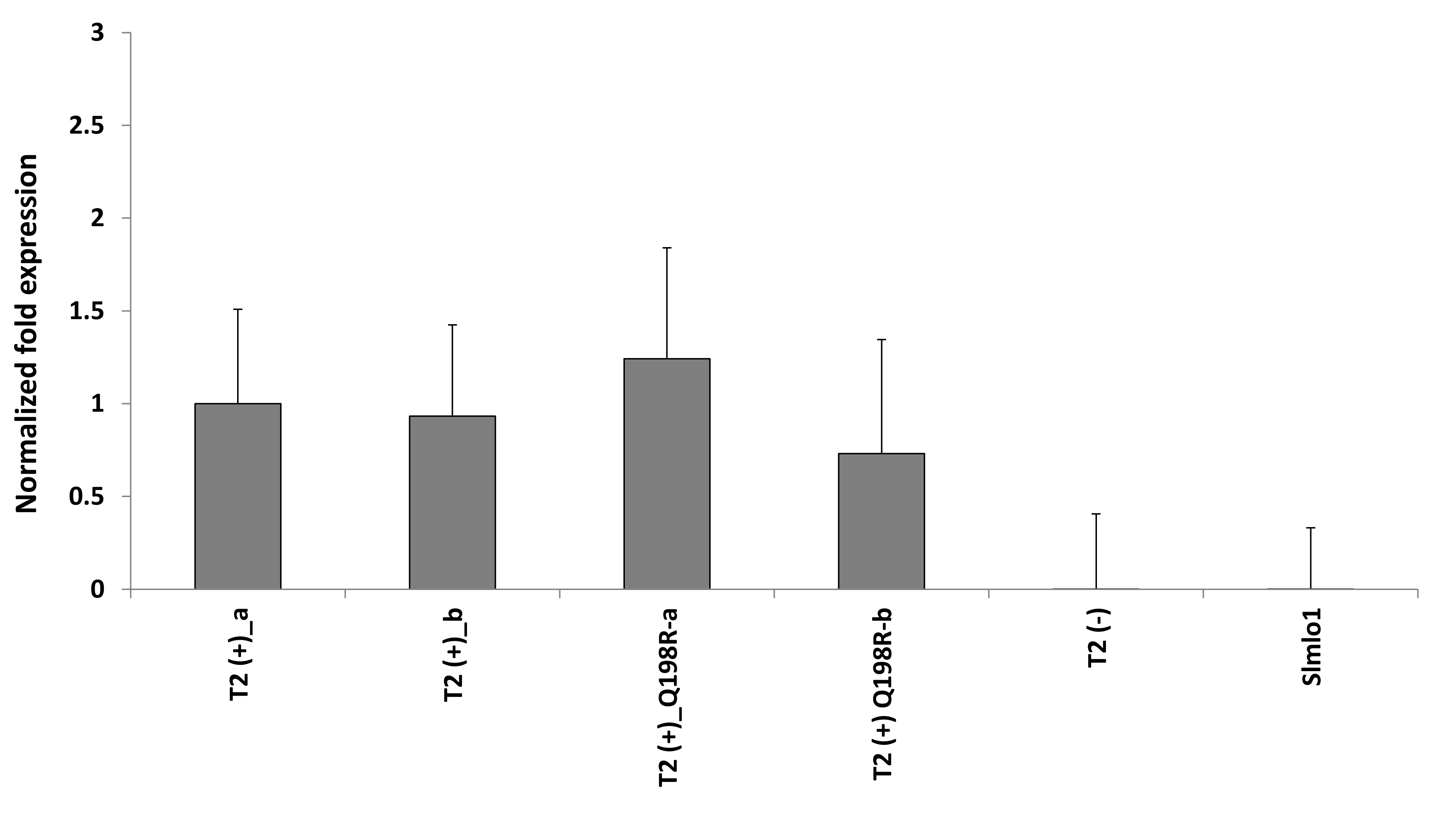


**Supplementary Table 1** Characteristics of the tobacco *NtMLO* gene family members identified in this study

| **Gene** | **SGN database sequence ID** | **Predicted TM** | **Amino-acid length** | **Clade** |
| --- | --- | --- | --- | --- |
| NtMLO1 | mRNA_127718_cds | 6 | 525 | V |
| NtMLO2 | mRNA_127185_cds | 6 | 532 | V |
| NtMLO3 | mRNA_63807_cds | 7 | 597 | V |
| NtMLO4 | mRNA_106507_cds | 6 | 605 | V |
| NtMLO5 | mRNA_52113_cds | 7 | 520 | V |
| NtMLO6 | mRNA_44723_cds | 6 | 554 | III |
| NtMLO7 | mRNA_90912_cds | 7 | 489 | VI |
| NtMLO8 | mRNA_125509_cds | 7 | 555 | III |
| NtMLO9 | mRNA_33476_cds | 7 | 455 | III |
| NtMLO10 | mRNA_91715_cds | 7 | 410 | III |
| NtMLO11 | mRNA_52133_cds | 7 | 492 | II |
| NtMLO12 | mRNA_46569_cds | 7 | 490 | II |
| NtMLO13 | mRNA_79933_cds | 6 | 508 | II |
| NtMLO14 | mRNA_23316_cds | 7 | 505 | II |
| NtMLO15 | mRNA_44406_cds | 7 | 558 | I |

**Supplementary Table 2** Characteristics of the potato *StMLO* gene family members identified in this study

| **Gene** | **Spud DB database**  **sequence ID** | **Chr.** | **Position from – to (bp)** | **Predicted introns** | **Predicted TM** | **Amino-acid length** | **Clade** |
| --- | --- | --- | --- | --- | --- | --- | --- |
| StMLO1^*^ | - | - | - | - | 6 | 519 | V |
| StMLO2 | PGSC0003DMG400013720 | 8 | 8,453,442-8,457,924 | 11 | 6 | 517 | II |
| StMLO3 | PGSC0003DMG400018975 | 10 | 17,809,877-17,818,901 | 13 | 3 | 456 | I |
| StMLO4 | PGSC0003DMG400020286 | 9 | 36,181,654-36,187,569 | 13 | 7 | 477 | III |
| StMLO5 | PGSC0003DMG400003574 | 2 | 39,242,676-39,247,920 | 13 | 4 | 455 | I |
| StMLO6 | PGSC0003DMG400012451 | 7 | 53,305,494-53,314,628 | 14 | 7 | 565 | I |
| StMLO7 | PGSC0003DMG400013667 | 2 | 38,189,087-38,196,233 | 13 | 7 | 552 | III |
| StMLO8 | PGSC0003DMG400018271 | 1 | 79,235,994-79,239,439 | 12 | 5 | 414 | II |
| StMLO9 | PGSC0003DMG400020605 | 3 | 36,041,611-36,048,004 | 13 | 6 | 366 | V |
| StMLO10 | PGSC0003DMG400023159 | 2 | 18,902,752-18,910,333 | 11 | 7 | 550 | III |
| StMLO11 | PGSC0003DMG400027665 | 6 | 57,855,801-57,859,958 | 12 | 7 | 507 | II |
| StMLO12 | PGSC0003DMG400030134 | 6 | 9,616,811-9,623,870 | 13 | 7 | 589 | V |
| StMLO13 | PGSC0003DMG400033623 | 8 | 42,249,518-42,256,102 | 14 | 7 | 532 | III |

* Features of StMLO1 refer to the homolog identified by a PCR-based approach for which no corresponding sequence is found in Spud DB database .

**Supplementary Table 3** Features and distribution of motifs conserved in the potato StMLO and tobacco NtMLO protein families, as predicted by the MEME software package. Correspondences with the motifs previously characterized by Deshmukh et al. (2014) in the soybean MLO protein family are reported.

|  | **Width** | **e-value** | **StMLO1** | **StMLO2** | **StMLO3** | **StMLO4** | **StMLO5** | **StMLO6** | **StMLO7** | **StMLO8** | **StMLO9** | **StMLO10** | **StMLO11** | **StMLO12** | **StMLO13** | **Sequence motif** | **Corresponding motif number in soybean (Deshmukh *et al.* 2014)** |
| --- | --- | --- | --- | --- | --- | --- | --- | --- | --- | --- | --- | --- | --- | --- | --- | --- | --- |
| **MOTIF 1** | 70 | 1.6e-397 | √ | √ | √ | √ | √ | √ | √ | √ | √ | √ | √ | √ | √ | PTWAVAVVCTVIVAISLAIERIIHKLGKWLKKKNKKALYEALEKIKEELMLLGFISLLLTVLQSYISKIC | 4 |
| **MOTIF 2** | 70 | 8.4e-376 | √ | √ | √ | √ | √ | √ | √ | √ | - | √ | √ | √ | √ | LIHFILFQNAFEIAFFFWIWWEYGFKSCFHDNFGFIIIRLVIGVIVQFLCSYSTLPLYALVTQMGSHMKK | 3 |
| **MOTIF 3** | 70 | 1.6e-295 | √ | √ | - | √ | - | √ | √ | - | √ | √ | √ | √ | √ | KFDFQKYIKRSLEDDFKVVVGISPVLWGFVVLFLLLNVHGWHAYFWIAFIPLIIILAVGTKLQHVITQMA | 1 |
| **MOTIF 4** | 51 | 1.0e-245 | √ | √ | √ | √ | √ | √ | √ | √ | √ | √ | √ | √ | √ | GKVPLLSLEALHQLHIFIFVLAVFHVLYSAITMALGGLKIRQWKxWEDEIK | 2 |
| **MOTIF 5** | 55 | 1.4e-176 | √ | √ | - | √ | - | √ | √ | √ | √ | √ | √ | √ | √ | RFTHETSFGRRHxSFWTKSPILFWIVCFFRQFFRSVxKSDYLTLRHGFIMAHLAP | 5 |
| **MOTIF 6** | 40 | 3.9e-038 | √ | √ | √ | √ | √ | √ | √ | - | - | √ | √ | √ | √ | SIFDEQVQKALHGWHKKAKKRRGHKxxRSxTTxSTSSSx | - |
| **MOTIF 7** | 40 | 2.00e-11 | - | - | - | √ | - | - | √ | - | - | √ | - | - | - | PESVADTLLPCPAKNKAAAEEEHRRRLLWEERRILAGAEP | - |

|  | **Width** | **e-value** | **NtMLO1** | **NtMLO2** | **NtMLO3** | **NtMLO4** | **NtMLO5** | **NtMLO6** | **NtMLO7** | **NtMLO8** | **NtMLO9** | **NtMLO10** | **NtMLO11** | **NtMLO12** | **NtMLO13** | **NtMLO14** | **NtMLO15** | **Sequence motif** | **Corresponding motif number in soybean (Deshmukh *et al*. 2014)** |
| --- | --- | --- | --- | --- | --- | --- | --- | --- | --- | --- | --- | --- | --- | --- | --- | --- | --- | --- | --- |
| **MOTIF 1** | 70 | 1.1e-580 | √ | √ | √ | √ | √ | √ | √ | √ | √ | √ | √ | √ | √ | √ | √ | PSDDLFWFNRPQLVLFLIHFVLFQNAFQLAFFFWIWYEYGLKSCFHDNVEDIIIRLVMGVGIQFLCSYIT | 3 |
| **MOTIF 2** | 70 | 3.9e-578 | √ | √ | √ | √ | √ | √ | √ | √ | √ | √ | √ | √ | √ | √ | √ | PTWAVAAVCFVIVAISIAIERIIHKLGKWLKKKHKKALYEALEKIKAELMLLGFISLLLTVSQYPISKIC | 4 |
| **MOTIF 3** | 70 | 5.4e-559 | √ | √ | √ | √ | √ | √ | √ | √ | √ | √ | √ | √ | √ | √ | √ | KFDFQKYIKRSLEDDFKVVVGISPPLWVFVVLFLLLNVHGWHAYFWIAFIPLIIILAVGTKLQHVITQMA | 1 |
| **MOTIF 4** | 56 | 3.5e-408 | √ | √ | √ | √ | √ | √ | √ | √ | √ | √ | √ | √ | √ | √ | √ | PERFRFTRETSFGRRHLSFWTRSPILLWIGCFFRQFFRSVSKSDYLTLRHGFIMAH | 5 |
| **MOTIF 5** | 56 | 1.3e-398 | √ | √ | √ | √ | √ | √ | √ | √ | √ | √ | √ | √ | √ | √ | √ | KGKVALISLDALHQLHIFIFVLAVLHVLYSALTMALGRAKIRGWKAWEDETQTHEY | 2 |
| **MOTIF 6** | 70 | 8.7e-246 | √ | √ | √ | √ | √ | √ | √ | √ | - | - | √ | √ | √ | √ | √ | LPLYALVTQMGSSMKKTIFDEHVATALKGWHHAAKKKKKLGGKHSNTTTGSSSPPATPGSQMEIIHLLRG | - |
| **MOTIF 7** | 40 | 8.30e-48 | - | - | - | - | - | √ | - | √ | √ | √ | √ | √ | √ | √ |  | IPESVANTMLPCPADEKxNETDEAEGHRRLLFELHRRGLA | - |
